# Modulating electron density of vacancy site by single Au atom for effective CO_2_ photoreduction

**DOI:** 10.1038/s41467-021-21925-7

**Published:** 2021-03-15

**Authors:** Yuehan Cao, Lan Guo, Meng Dan, Dmitry E. Doronkin, Chunqiu Han, Zhiqiang Rao, Yang Liu, Jie Meng, Zeai Huang, Kaibo Zheng, Peng Chen, Fan Dong, Ying Zhou

**Affiliations:** 1 State Key Laboratory of Oil and Gas Reservoir Geology and Exploitation, Southwest Petroleum University, Chengdu, China; 2 The Center of New Energy Materials and Technology, School of New Energy and Materials, Southwest Petroleum University, Chengdu, China; 3 The Division of Chemical Physics and Nano-Lund, Lund University, Lund, Sweden; 4 Institute for Chemical Technology and Polymer Chemistry and Institute of Catalysis Research and Technology, Karlsruhe Institute of Technology, Karlsruhe, Germany; 5 Department of Chemistry, Technical University of Denmark, Kongens Lyngby, Denmark; 6 Research Center for Environmental Science and Technology, Institute of Fundamental and Frontier Sciences, University of Electronic Science and Technology of China, Chengdu, China

**Keywords:** Catalyst synthesis, Artificial photosynthesis, Photocatalysis

## Abstract

The surface electron density significantly affects the photocatalytic efficiency, especially the photocatalytic CO_2_ reduction reaction, which involves multi-electron participation in the conversion process. Herein, we propose a conceptually different mechanism for surface electron density modulation based on the model of Au anchored CdS. We firstly manipulate the direction of electron transfer by regulating the vacancy types of CdS. When electrons accumulate on vacancies instead of single Au atoms, the adsorption types of CO_2_ change from physical adsorption to chemical adsorption. More importantly, the surface electron density is manipulated by controlling the size of Au nanostructures. When Au nanoclusters downsize to single Au atoms, the strong hybridization of Au 5*d* and S 2*p* orbits accelerates the photo-electrons transfer onto the surface, resulting in more electrons available for CO_2_ reduction. As a result, the product generation rate of Au_SA_/Cd_1−x_S manifests a remarkable at least 113-fold enhancement compared with pristine Cd_1−x_S.

## Abstract (web summary)

The electron density of reactive sites significantly affects catalytic performances. Here, authors demonstrate the electron density of different reactive sites can be modulated by regulating the type of vacancy and the size of Au, leading to effective CO_2_ photoreduction.

## Introduction

As a representative greenhouse gas, carbon dioxide (CO_2_) has brought out serious environmental problems needing to solve urgently^1,2^. Photocatalytic CO_2_ conversion technology has attracted extensive interest, since the conversion of CO_2_ into high value-added chemicals could potentially be achieved under relatively mild conditions, which facilitates its resource utilization^3,4^. Generally, photocatalytic CO_2_ conversion is a typical photoreduction reaction, which needs electrons to participate in^5–7^. Different from other photoreduction reactions, such as hydrogen evolution reaction (two electrons), photocatalytic CO_2_ conversion is an inherently multi-electron reduction process^8–10^. To this end, photocatalysts are required to offer abundant electrons for the CO_2_ molecule to promote its transformation in the reaction process^11^.

Previous studies suggest that metals, especially noble metals, have been commonly introduced to enhance the surface electron density of photocatalysts^12,13^. It is generally agreed that the photocatalytic performance is greatly dependent on the metal center and its local coordination. Since the local electron density will be redistributed after introducing metal and the majority of electrons are accumulated around the metal and its surrounding atoms^14,15^, many efforts have focused on changing the metal center and/or ligands to achieve high efficiency for CO_2_ photoreduction. With in-depth research, it is further found that electron transfer is tremendously dependent on the size of metal nanostructures or the type of local coordination^16,17^. For instance, Wu et al. reported that the electrons have transferred from single Pt atoms to sulfur-doped carbon support and the direction of electron transfer will be reversed after loading Pt nanoclusters^16^. Meanwhile, Dai et al. found that the types of vacancies (boron or nitrogen vacancies) could affect the electron transfer direction between Pt nanoparticles and BN nanosheets^17^. Hence, it is speculated that the electrons may be localized at other sites instead of metal nanostructures. Indeed, previous studies suggest that the interaction of adsorbed CO_2_ molecule and metal site is relatively weak since the formed metal–C or metal–O bonds are weaker than the highly stable C–O bonds in CO_2_ molecule^8,18,19^. This will lead to the easy cleaving of metal–C or metal–O bonds during the reaction process, hindering the further transformation of CO_2_^8^. The above considerations suggest that controlling the size of metal nanostructures and/or the type of vacancies might realize the accumulation of electrons at other sites instead of metal nanostructures, which is likely to promote the adsorption and conversion of CO_2_.

**Fig. 1:**
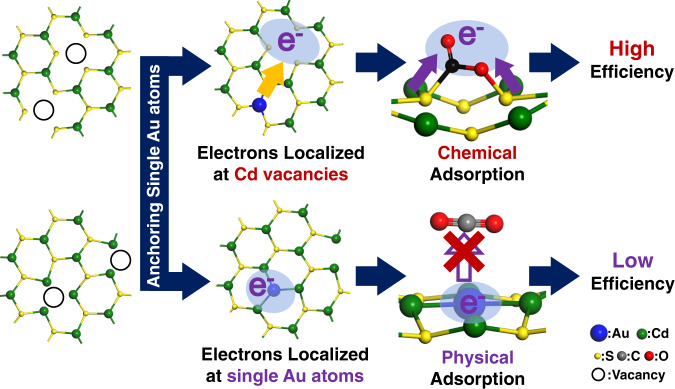
Photocatalytic mechanism. Schematic illustration of single Au atoms in CdS to promote CO_2_ photoreduction.

For this purpose, CdS is selected to be a model material due to the easy-fabricated vacancies and favorable solar light response^20–22^. According to the pre-experiments in Supplementary Fig. 1, Au is chosen as the dopant metal^23–25^. We have designed and fabricated a series of Au-anchored CdS photocatalysts and reported a conceptually different mechanism for modulating surface electron density to promote the conversion of CO_2_. As displayed in Fig. 1, when the type of vacancies changes from S vacancies to Cd vacancies, the direction of electron transfer will be reversed after anchoring single Au atoms, resulting in the accumulation of electrons in neighboring Cd vacancies instead of single Au atoms. Benefiting from this, CO_2_ could be chemically bonded on the Cd vacancies instead of physical adsorption on single Au atoms to promote its effective activation.

## Results

### Catalyst characterization

**Fig. 2:**
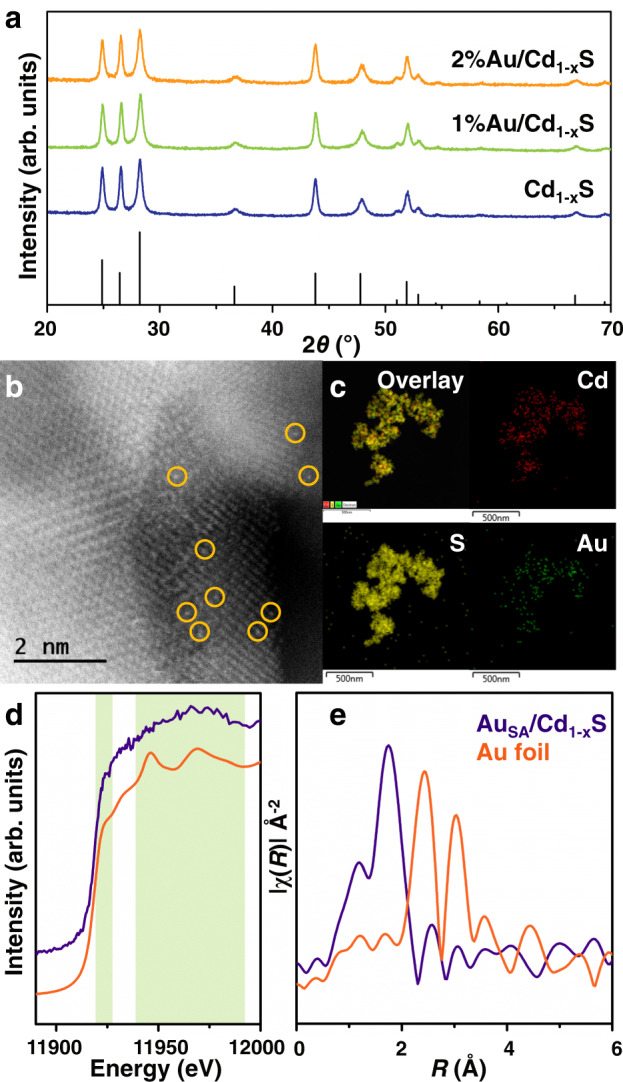
Characterizations. **a** XRD patterns of Cd_1-x_S, 1%Au/Cd_1-x_S, and 2%Au/Cd_1-x_S samples. **b** HAADF-STEM image of 1%Au/Cd_1-x_S, single atomic Au sites high-lighted by yellow circles. **c** Elemental mappings of 1%Au/Cd_1-x_S. **d** XAFS analysis of Au_SA_/Cd_1-x_S: Au L_3_ XANES spectra of the sample and the reference foil. **e** Corresponding k^1^-weighted Fourier transform (FT) EXAFS spectra.

Based on the above considerations, the CdS with Cd vacancies (Cd_1−x_S) is first prepared by the hydrothermal method. X-ray diffraction (XRD) pattern and transmission electron microscopy (TEM) images (cf. Fig. 2a and Supplementary Fig. 2a, b) of the synthesized sample display that hexagonal CdS with the nanoparticle morphology is fabricated. Meanwhile, the electron paramagnetic resonance (EPR) signals with the *g* factor of 1.99 and 2.03 are observed for the synthesized sample (cf. Supplementary Fig. 3), which are ascribed to sulfur atoms surrounding Cd vacancies in hexagonal CdS^26–28^. According to the element analysis, the ratios of Cd and S are quantitated as 0.76:1 (cf. Supplementary Table 1). The above results confirm the existence of Cd vacancies in the synthesized sample. After Au loading, XRD patterns of both 1%Au/Cd_1−x_S and 2%Au/Cd_1−x_S samples have rarely changed compared with Cd_1−x_S (cf. Fig. 2a). For 1%Au/Cd_1−x_S, there are no Au nanostructures observed in TEM and HRTEM images (cf. Supplementary Fig. 2c, d). Hence, to reveal the atomic-resolution structure of 1%Au/Cd_1−x_S sample, aberration-corrected high-angle annular dark-field scanning TEM is shown in Fig. 2b. The bright spots corresponding to heavy Au atoms (marked by yellow circles) are homogeneously dispersed, indicating that the Au atoms are atomically dispersed on the Cd_1−x_S. Meanwhile, the element mapping of the 1%Au/Cd_1−x_S (cf. Fig. 2c) shows the uniform distribution of Au, Cd, and S elements. With the increase of loading content, some nanoparticles are observed in TEM and HRTEM images of 2%Au/Cd_1−x_S sample (cf. Supplementary Fig. 2e, f). The interplanar distance in these nanoclusters is ca 1.15 Å corresponding to the (222) planes of metallic Au^29^. This is indicated that part of single Au atoms is aggregated together to form the Au nanoclusters with the increase of loading content. According to the size distributions of Au nanoclusters and element mapping in Supplementary Fig. 4, the Au nanoclusters are homogeneously distributed on Cd_1−x_S and the sizes of Au nanoclusters are mainly in the range of 1–3 nm. The above results indicate that the single Au atoms (Au_SA_) and Au clusters (Au_NC_) are successfully introduced in the 1%Au/Cd_1−x_S and 2%Au/Cd_1−x_S samples, respectively. To distinguish Au nanostructures in different samples, 1%Au/Cd_1−x_S and 2%Au/Cd_1−x_S samples are referred as Au_SA_/Cd_1−x_S and Au_NC_/Cd_1−x_S for simplicity in the following study.

To investigate the coordination environments of Au in the Au_SA_/Cd_1−x_S sample, Au L_3_-edge X-ray absorption near-edge structure (XANES) and extended X-ray absorption fine structure (EXAFS) spectra are presented in Fig. 2d, e. The white line peak of Au_SA_/Cd_1−x_S sample is different from that of the reference Au foil. The EXAFS spectrum shows that Au_SA_/Cd_1−x_S exhibits a peak near 1.7 Å without any significant Au–Au contribution between 2 and 4 Å, confirming the atomic dispersion of Au on Cd_1−x_S. The quantitative structural parameters of Au in the Au_SA_/Cd_1−x_S (cf. Supplementary Table 2) demonstrate that one Au atom is bonded with two or three S atoms to form the Au–S bonds. To further identify the loading position of single Au atoms, EPR spectra are carried out in Supplementary Fig. 5. The intensities of EPR signals assigned to Cd vacancies decreased after introducing single Au atoms, indicating that part of Cd vacancies are covered by single Au atoms. Furthermore, the models of Au-loaded Cd_1−x_S surface with different sites (surface S and Cd vacancy) are constructed by density functional theory (DFT) method (cf. Supplementary Table 3). The calculated formation energy of single Au atom anchored on Cd vacancy is −5.30 eV, which is much more negative than that on surface S atoms (*E*_*f*_ = −1.89 eV). It is hence suggested that single Au atoms are anchored on the Cd vacancies to form the Au–S bonds in the Au_SA_/Cd_1−x_S sample.

Similarly, the CdS with S vacancies (CdS_1−x_) is synthesized by the hydrothermal method (cf. Supplementary Fig. 6). There are no EPR signals assigned to S vacancies observation in the synthesized sample (cf. Supplementary Fig. 7). To confirm the existence of S vacancies, the Cd K-edge XANES and EXAFS spectra of CdS_1−x_ and pristine CdS (CdS) samples are shown in Supplementary Fig. 8. The white line peak of the CdS_1−x_ is similar to that of the CdS and both of them are different from that of the reference Cd foil. According to the EXAFS spectra, the CdS sample shows bulk crystalline CdS. In the case of the CdS_1−x_ sample, intensity of the first coordination shell is lower than that of CdS, implying fewer S-neighbors than in the bulk CdS. Combined with the element analysis, the ratio of Cd and S are quantitated as 1:0.73 (cf. Supplementary Table 1). It is confirmed that there are S vacancies in the CdS_1−x_ sample.

Furthermore, the same impregnation method is employed to synthesize the 1%Au/CdS_1−x_ and 2%Au/CdS_1−x_ (cf. Supplementary Fig. 6). The XANES and EXAFS spectra of CdS_1−x_ and 1%Au/CdS_1−x_ are shown in Supplementary Fig. 9 and 10. It is found that the intensity of the first coordination shell in CdS_1−x_ sample increases after loading Au, suggesting that part of S vacancies are filled with Au (cf. Supplementary Fig. 9). Meanwhile, in the case of 1%Au/CdS_1−x_, there are no significant peaks assigned to Au–Au bonds found in the range of 2–4 Å, confirming the atomic dispersion of Au on CdS_1−x_ (cf. Supplementary Fig. 10). The above results suggest that single Au atoms are anchored on the S vacancies. Hence, the 1%Au/CdS_1−x_ sample is referred to Au_SA_/CdS_1−x_.

In brief, the CdS samples with different types of vacancies (Cd and S vacancies) are successfully fabricated and the introduced single Au atoms are anchored on the vacancies. In the case of the Au-anchored Cd_1−x_S systems, with the increase of loading content, Au has changed from single atoms into nanoclusters.

### Photocatalytic CO_2_RR measurement

**Fig. 3:**
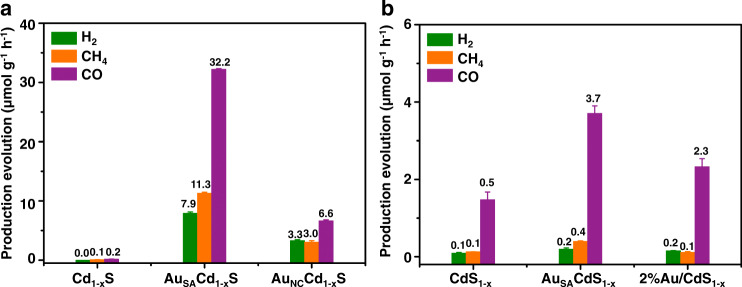
The photocatalytic performance of CO_2_RR. Average yield in the photocatalytic conversion of CO_2_ with H_2_O in the presence of water vapor under UV-visible light. **a** Over Cd_1-x_S, Au_SA_/Cd_1-x_S, and Au_NC_/Cd_1-x_S samples. **b** Over CdS_1-x_, Au_SA_/CdS_1-x_, and 2%Au/CdS_1-x_ samples. The error bar was drawn based on the calculated standard error of two parallel tests.

As depicted in Fig. 3 and Supplementary Fig. 11, photocatalytic CO_2_ reduction tests are carried out to evaluate the effect of Au on photoreduction efficiency. Cd_1−x_S shows low CO and CH_4_ generation rates of 0.2 and 0.1 μmol g^−1^ h^−1^, respectively. After anchoring Au nanoclusters, the product generation rates are enhanced. It is interesting that with the introduction of single Au atoms, the CO, CH_4_, and H_2_ generation rates are highly promoted to 32.2, 11.3, and 7.9 μmol g^−1^ h^−1^, respectively. It manifests a remarkable 161-fold and 113-fold enhancement over Cd_1−x_S as well as 5-fold, 4-fold, and 2-fold enhancement compared with Au_NC_/Cd_1−x_S. For CdS_1−x_ systems (cf. Fig. 3b), the introduction of Au brings out an enhancement of CO_2_ photoreduction efficiency as well. Among all the samples, Au_SA_/CdS_1−x_ reveals the highest CO_2_ photoreduction efficiency with the CO, CH_4_, and H_2_ generation rates of 3.7, 0.4, and 0.2 μmol g^−1^ h^−1^, which is a 2-fold, 4-fold, and 2-fold enhancement compared over the pristine CdS_1−x_. The above results indicate that, compared with S vacancies, single Au atoms bring much more significant improvement for CO_2_ photoreduction efficiency when they are anchored on Cd vacancies. Meanwhile, when Au changes from single atoms into nanoclusters, the CO_2_ photoreduction efficiency is gradually decreased. Furthermore, the photocatalytic CO_2_RR performance of Au_SA_/Cd_1−x_S is relatively satisfactory compared with that of other metal sulfide, CdS-based, and noble-metal based photocatalysts in the published works (cf. Supplementary Table 4)^8,30–38^. To confirm that the CO and CH_4_ products are generated from the CO_2_ conversion driven by Au_SA_/Cd_1−x_S, the isotope labeling is conducted using ^13^CO_2_ as the reactant for qualitative analysis. Supplementary Fig. 12 displays that the generated CO and CH_4_ originate from the CO_2_ conversion driven by Au_SA_/Cd_1−x_S sample instead of desorption of adsorbed species from the surface. Besides, the peak at m/z = 32 is detected, which is assigned to generated oxidation product O_2_. With a comparison of XRD, TEM, and HRTEM images between fresh and used Au_SA_/Cd_1−x_S samples (cf. Supplementary Fig. 13 and 14), the single Au atoms is not aggregated during the reaction process, indicating good stability.

## Discussion

### CO_2_ adsorption during photocatalysis

To uncover the main factors of enhanced photocatalytic CO_2_ reduction performance, solar light absorption is first investigated by UV-vis spectroscopy (cf. Supplementary Fig. 15). The absorption edges of Au_SA_/Cd_1−x_S and Au_NC_/Cd_1−x_S have barely changed compared with Cd_1−x_S. For the CdS_1−x_ system, the introduction of Au does not change the absorption edge as well. This suggests that solar light absorption is not the determining factor in the photocatalytic CO_2_ reduction performance. After light illumination, the excited electrons will finally transfer to the surface of the catalyst and combine with the adsorbed species to promote their conversion. The conversion of CO_2_ involves the following steps. The adsorption of CO_2_ on the surface of photocatalyst is the prerequisite step, which is relatively difficult due to the closed-shell electronic configuration of CO_2_. Then, adsorbed CO_2_ will obtain electrons from the surface to generate the carbon active species (e.g., CO_2_^−^, HCO_3_^−^, CO_3_^2−^). These first two steps are aimed to activate the C–O bond of CO_2_ molecule. Finally, these species will be further transformed into the desired products (e.g., CO, CH_4_), which need abundant electrons to take part in to promote their effective transformation. Hence, the adsorption behavior of CO_2_ not only determines the occurrences of reaction, but significantly affects the conversion efficiency.

**Fig. 4:**
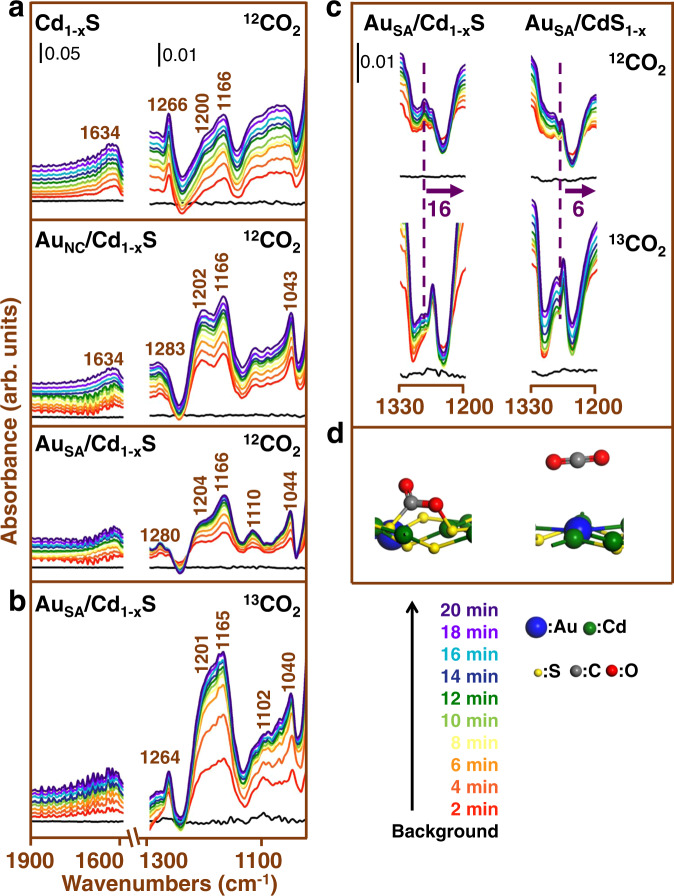
In situ DRIFTS measurement. **a** In situ DRIFTS spectra of the ^12^CO_2_ adsorption process over Cd_1-x_S, Au_NC_/Cd_1-x_S, and Au_SA_/Cd_1-x_S. **b** In situ DRIFTS spectra of the ^13^CO_2_ adsorption process over Au_SA_/Cd_1-x_S. **c** The comparison between in situ DRIFTS spectra of ^12^CO_2_ and ^13^CO_2_ adsorption process over Au_SA_/Cd_1-x_S and Au_SA_/CdS_1-x_. **d** The adsorption models of CO_2_ on different surfaces on single Au atom in Au_SA_/CdS_1-x_ and on Cd vacancy in Au_SA_/Cd_1-x_S.

Based on these considerations, in situ diffuse reflectance infrared Fourier transform spectroscopy (DRIFTS) is performed to detect the generated intermediates during the CO_2_ adsorption process on the surfaces of Cd_1−x_S and CdS_1−x_ systems (cf. Fig. 4 and Supplementary Fig. 16). For pristine Cd_1−x_S (cf. Fig. 4a), there are some absorption bands assigned to bicarbonate (HCO_3_^−^; *δ*(COH): 1200 and 1166 cm^−1^), monodentate carbonate (m-CO_3_^2−^; *ν*(C–O): 1140–1030 cm^−1^) and carboxylate (CO_2_^−^; *ν*(O–C–O)_s_: 1266 cm^−1^) detected with the prolonged time of adsorption^39^. Meanwhile, the band assigned to COOH* is detected at 1634 cm^−1^,^40,41^ suggesting that the adsorbed CO_2_ is combined with H* and partly converted to the COOH*. After anchoring Au clusters, the intensities of absorption bands assigned to HCO_3_^−^, CO_2_^−^, and COOH* have decreased. There is a new band appearing at 1043 cm^−1^, which is assigned to HCO*^42,43^. This indicates that part of the formed COOH* is converted to HCO*. Different from it, some of the absorption bands (HCO_3_^−^ and COOH*) almost disappear over Au_SA_/Cd_1−x_S. It should be noted that there is a new band detected at 1110 cm^−1^ assigned to H_3_CO*^44,45^. The intensity of the absorption bands assigned to HCO* is much lower than that over Au_NC_/Cd_1−x_S, revealing that the majority of formed COOH* are quickly converted into HCO* and then transform into H_3_CO*. The conversion pathway of CO_2_ over different samples in Au/Cd_1−x_S systems during the adsorption process could be deduced as follows:

$${\mathrm{CO}}_2^ \ast \to {\mathrm{CO}}_2^ - /{\mathrm{HCO}}_3^ - /{\mathrm{CO}}_3^{2 - } \to {\mathrm{COOH}}^ \ast \quad \quad {\mathrm{over}}\;{\mathrm{Cd}}_{1 - {\mathrm{x}}}{\mathrm{S}} \;\;$$

$${\mathrm{CO}}_2^ \ast \to {\mathrm{CO}}_2^ - /{\mathrm{HCO}}_3^ - /{\mathrm{CO}}_3^{2 - } \to {\mathrm{COOH}}^ \ast \to {\mathrm{CO}}^ \ast \to {\mathrm{HCO}}^ \ast \to {\mathrm{H}}_2{\mathrm{CO}}^ \ast \to {\mathrm{H}}_3{\mathrm{CO}}^ \ast \quad {\mathrm{over}}\;{\mathrm{Au}}_{{\mathrm{SA}}}/{\mathrm{Cd}}_{1 - {\mathrm{x}}}{\mathrm{S}}$$

$${\mathrm{CO}}_2^ \ast \to {\mathrm{CO}}_2^ - /{\mathrm{HCO}}_3^ - /{\mathrm{CO}}_3^{2 - } \to {\mathrm{COOH}}^ \ast \to {\mathrm{CO}}^ \ast \to {\mathrm{HCO}}^ \ast \quad \quad {\mathrm{over}}\;{\mathrm{Au}}_{{\mathrm{NC}}}/{\mathrm{Cd}}_{1 - {\mathrm{x}}}{\mathrm{S}}$$

It is indicated that the introduction of Au significantly promotes the further conversion of CO_2_ on Cd_1−x_S surface. When Au nanoclusters downsize to single Au atoms, the carbon active species can be deeply converted into H_3_CO*, which is beneficial for the subsequent reaction. Different from the Cd_1−x_S system, with the introduction of Au nanostructures, there are no absorption bands assigned to new intermediates appearing on CdS_1−x_ surface in the process of CO_2_ adsorption (cf. Supplementary Fig. 16). It could be concluded that the introduction of Au has a slight effect in the CO_2_ adsorption and conversion on CdS_1−x_ surface.

**Fig. 5:**
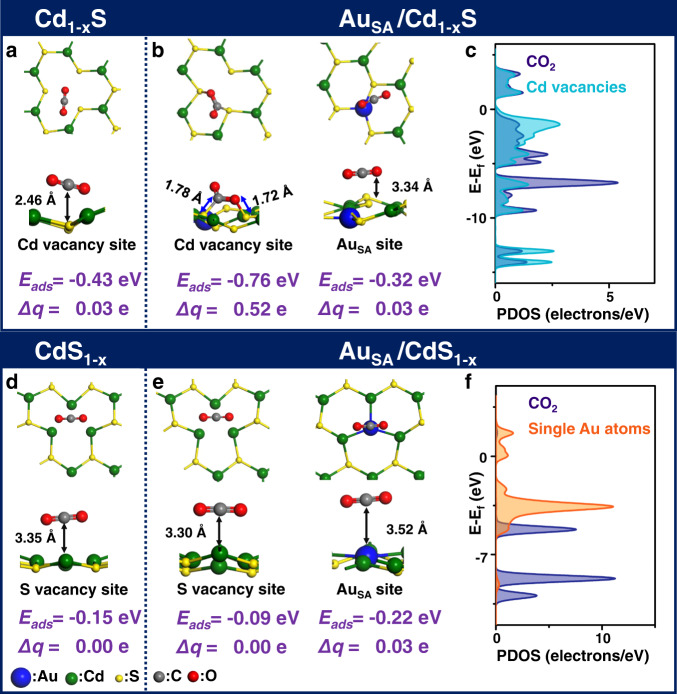
CO_2_ adsorption models on different surfaces. Configurations of CO_2_ adsorption**: a** on Cd_1-x_S; **b** Au_SA_/Cd_1-x_S; **d** CdS_1-x_; **e** Au_SA_/CdS_1-x_. PDOS of CO_2_ and adsorption site: **c**. in Au_SA_/Cd_1-x_S; **f** Au_SA_/CdS_1-x_.

The above discussions suggest that the types of vacancies and the size of Au nanostructures present a different effect on the intermediates in the CO_2_ adsorption process. In previous studies, reaction sites have possibly affected the generation of intermediates^46^. Based on it, Au nanostructures (Au site) and vacancies (vacancy site) are selected to be the different CO_2_ adsorption sites. The CO_2_ adsorption models on Au/Cd_1−x_S and Au/CdS_1−x_ are constructed by the DFT method (cf. Fig. 5 and Supplementary Fig. 17 and 18). The corresponding adsorption energies are summarized in Supplementary Table 5. For the pristine CdS_1−x_, CO_2_ will spontaneously adsorb on the S vacancies due to the negative adsorption energy. After anchoring single Au atoms, the adsorption energies change slightly compared with that of pristine CdS_1−x_. According to the comparison of adsorption energies, CO_2_ prefers to adsorb on single Au atoms instead of S vacancies. Similarly, in the case of pristine Cd_1−x_S, CO_2_ will also spontaneously adsorb on the Cd vacancies (*E*_ads_ = −0.43 eV). With the introduction of single Au atoms, the adsorption energy has significantly decreased, implying that single Au atoms promotes the adsorption of CO_2_ on Cd_1−x_S surface. It should be noted that CO_2_ is more likely to adsorb on the Cd vacancies instead of single Au atoms due to the more negative adsorption energy. To further identify the adsorption site in Au_SA_/Cd_1−x_S, the different surfaces are probed by N_2_ sorption (cf. Supplementary Fig. 19). The specific surface area of Cd_1−x_S (25.4 m^2^/g) has increased after anchoring single Au atoms (33.9 m^2^/g). It is suggested that the introduction of single Au atoms enhances the amount of adsorption site. However, the CO_2_ adsorption capacity of Cd_1−x_S is decreased from 72.9 to 66.2 μmol g^−1^ (cf. Supplementary Fig. 20) after introducing single Au atoms. It is hence confirmed that the real adsorption sites of CO_2_ in Au_SA_/Cd_1−x_S should be the vacancies instead of single Au atoms. Furthermore, combined with in situ DRIFTS spectra, CO is used as the probe to identify the reactive reaction site of CO_2_ reduction on Au_SA_/Cd_1−x_S surface (cf. Supplementary Fig. 21). The absorption bands at 2170 and 2109 cm^−1^ are assigned to gaseous CO^47^. Furthermore, the absorption band assigned to C=O bond and S–O are detected on the surface (*v*(C=O): 1621 cm^−1^; *v*(S–O): 1209 and 1162 cm^−1^)^47–51^. It is suggested that the CO molecule adsorbs on the Cd vacancy sites and combines with the S atoms to form C=O–S bonds. Meanwhile, there are some absorption bands detected at 1100 and 1044 cm^−1^, which are assigned to HCO* and H_3_CO*. The above results indicate that when CO adsorbs on Cd vacancy, it will combine with H* to further convert into HCO* and H_3_CO*, which is in accordance with the observations in DRIFTS spectra of the CO_2_ adsorption process.

Furthermore, according to the partial density of states (PDOS) (cf. Fig. 5f), it is observed that the interaction between CO_2_ and single Au atoms in the Au_SA_/CdS_1−x_ is weak. Different from it, there is a strong interaction between CO_2_ and Cd vacancies in the Au_SA_/Cd_1−x_S (cf. Fig. 5c). More importantly, the C and O atoms of CO_2_ molecule are bonded with two S atoms of the surface to form the S–C and S–O bonds (cf. Fig. 5b). As a result, the electrons transfer from the surface to the bonded CO_2_ molecule (0.52 e) to promote its activation, resulting in C–O–C bond angle bending and elongation of both C–O bonds. To further prove the effect of different adsorption sites in the CO_2_ adsorption behavior, isotope labeling is carried out on Au_SA_/Cd_1−x_S and Au_SA_/CdS_1−x_ using ^13^CO_2_ instead of ^12^CO_2_ for the DRIFTS analysis (cf. Fig. 4b and Supplementary Fig. 16d). It is observed that, compared with using ^12^CO_2_ as reactants, similar absorption bands are detected on both Au_SA_/Cd_1−x_S and Au_SA_/CdS_1−x_ under ^13^CO_2_ atmosphere. Meanwhile, the positions of all the absorption bands have a ^13^C frequency shift toward low wavenumber in varying degrees (2–16 cm^−1^). In the process of ^12^CO_2_ adsorption (cf. Fig. 4c), there is an absorption band detected at 1280 cm^−1^ on both Au_SA_/Cd_1−x_S and Au_SA_/CdS_1−x_, which is assigned to the vibration of C–O bonds in CO_2_^−^ (adsorption state of CO_2_). In the process of ^13^CO_2_ adsorption, there is a small ^13^C frequency shift (6 cm^−1^) occurring on the surface of Au_SA_/CdS_1−x_. It should be noted that there is a large ^13^C frequency shift (16 cm^−1^) of this absorption band detected on the surface of Au_SA_/Cd_1−x_S. It is indicated that, compared with Au_SA_/CdS_1−x_ surface, the adsorbed CO_2_ obtains more electrons from Au_SA_/Cd_1−x_S surface to weaken the C–O bond, leading to a larger ^13^C frequency shift. Meanwhile, according to the value of ^13^C frequency shift, more than one C–O bonds might be bonded to the surface^11,52^. Based on the above discussions, it is speculated that CO_2_ molecule is physically adsorbed on single atoms of Au_SA_/CdS_1−x_ surface. The formation of bonds and effective electron transfer between surface and CO_2_ indicate that CO_2_ molecule is chemically adsorbed on Cd vacancies of Au_SA_/Cd_1−x_S surface. In brief, when the type of vacancies changes from S vacancies to Cd vacancies, the CO_2_ adsorption sites change from single Au atoms to the vacancies. Benefiting from this, CO_2_ could be chemically bonded on the Cd vacancies instead of physical adsorption on single Au atoms. The strong interaction between CO_2_ and adsorption sites (Cd vacancies) profits the formation of stable electron-transfer channels. As a result, CO_2_ could obtain more electrons from the adsorption sites to promote its deep transformation.

**Fig. 6:**
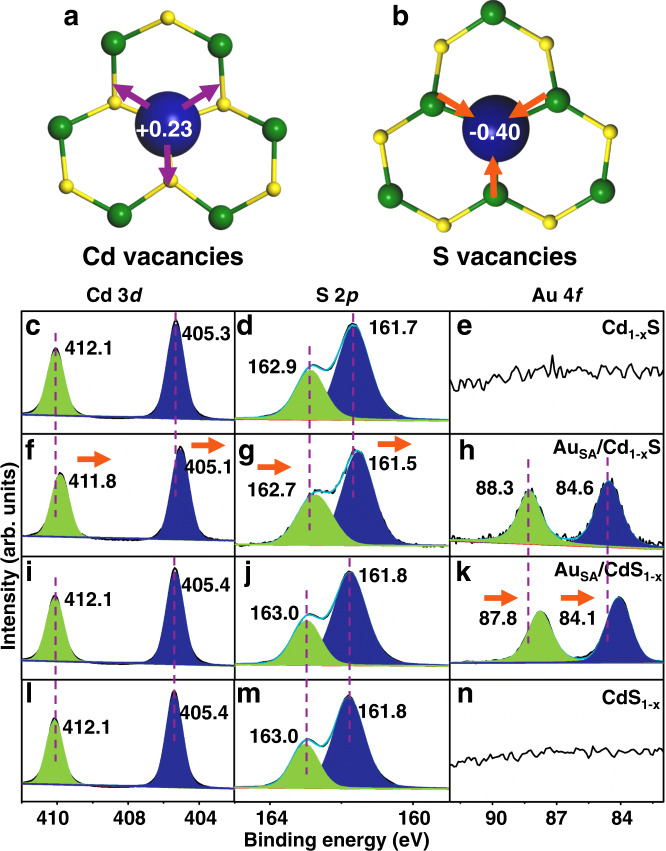
Charge transfer on different surfaces. Electron transfer between Au and CdS with different types of vacancies: **a** Cd vacancies; **b** S vacancies. XPS spectra: **c**–**e** Cd_1-x_S; **f**–**h** Au_SA_/Cd_1-x_S; **i**–**k** CdS_1-x_; **l**–**n** Au_SA_/CdS_1-x_ samples.

To identify the internal factors affecting adsorption sites, the charge population of Cd_1−x_S and CdS_1−x_ surfaces after anchoring Au nanostructures is explored by DFT calculations (cf. Fig. 6a and 6b). For the Au anchored on Cd vacancies, the electrons (0.23 e) will transfer from single Au atoms to Cd_1−x_S to enhance the surface electron density. More importantly, the majority of electrons are localized at the neighboring Cd vacancy site. On the contrary, the direction of electron transfer between Au and CdS_1−x_ is reversed, resulting in the localization of electrons at single Au atoms (0.40 e). To further identify the electron transfer between Au and Cd_1−x_S or CdS_1−x_, X-ray photoelectron spectroscopy (XPS) spectra are presented in Fig. 6. For Cd_1−x_S system (cf. Fig. 6c–h), the peaks indexed to the Cd–S bond in Cd 3*d* and S 2*p* spectra have a shift toward low binding energy (0.2–0.3 eV) compared with the pristine Cd_1−x_S, indicating that Cd_1−x_S receives electrons from the single Au atoms. While for CdS_1−x_ system (cf. Fig. 6i–n), the peaks corresponding to the Cd–S bond barely change after anchoring single Au atoms. It should be noted that the peaks in Au 4*f* spectra of Au_SA_/CdS_1−x_ system are located at 87.8 and 84.1 eV and the binding energies are much lower (c.a. 0.5 eV) than that of Au_SA_/Cd_1−x_S. This suggests that the electrons are localized at single Au atoms instead of CdS_1−x_ in Au_SA_/CdS_1−x_ system. To identify the internal factors affecting the direction of electron transfer between single Au atoms and Cd_1−x_S or CdS_1−x_, the potential energy plots are given in Supplementary Fig. 22. In the case of Au_SA_/CdS_1−x_, the potential energy of CdS_1−x_ is much lower than that of single Au atoms. Benefiting from it, there is a driving force promoting electron transfer from CdS_1−x_ to single Au atoms. Opposite to that, the potential energy of single Au atoms is lower than that of Cd_1−x_S, resulting in the reversal of electron transfer direction. Hence, it is observed that the electrons are localized at the single Au atoms when they anchor on S vacancies. After single Au atoms anchored on Cd vacancies, the electrons will conversely transfer from single Au atoms to Cd_1−x_S and accumulate on Cd vacancies.

### Photo-induced carrier transfer during photocatalysis

**Fig. 7:**
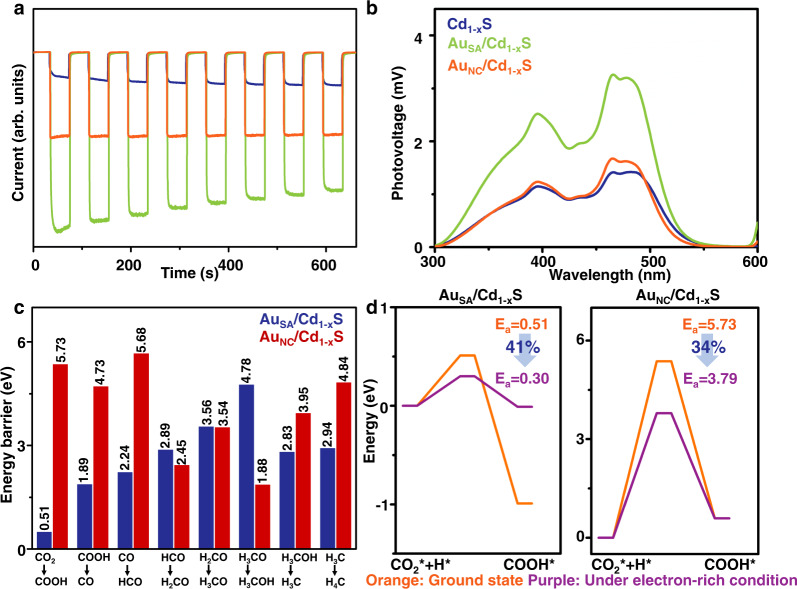
Photo-induced carrier transfer and energy barriers in the process of CO_2_RR based on DFT calculations. **a** Transient photocurrent response and **b** SPV spectra of Cd_1-x_S, Au_SA_/Cd_1-x_S, and Au_NC_/Cd_1-x_S samples. **c** Energy barriers of CO_2_ reduction on Au_SA_/Cd_1-x_S and Au_NC_/Cd_1-x_S. **d** Reaction profiles of CO_2_ transition into COOH on Au_SA_/Cd_1-x_S and Au_NC_/Cd_1-x_S.

According to the DFT and XPS spectra (cf. Supplementary Fig. 23–25), it is found that with the increased content of Au, the electron transfer is gradually weakened for both Cd_1−x_S and CdS_1−x_ systems. Hence, to further identify the underlying roles of Au content on the surface electron density, the Au/Cd_1−x_S system is selected as the representative in the following discussion. In general, electron transfer might be related to the local electronic structure. According to PDOS in Supplementary Fig. 26, compared with the Au_NC_/Cd_1−x_S, the hybridization between Au 5*d* and S 2*p* orbits in Au–S bonds is much stronger for Au_SA_/Cd_1−x_S. As a result, more electrons are transferring from Au 5*d* orbits in single Au atoms to Cd_1−x_S compared with Au clusters (cf. Supplementary Fig. 27). This is identified by XPS analysis in Supplementary Fig. 25. The peaks indexed to the Cd–S bond in Cd 3*d* and S 2*p* spectra for Au_SA_/Cd_1−x_S have a shift toward low binding energy (0.2–0.3 eV) and the Au 4*f* spectrum shifts to high binding energy (0.4–0.6 eV) compared with Au_NC_/Cd_1−x_S. It is indicated that the interaction between single Au atoms and Cd vacancies is much stronger than that with Au clusters. Benefiting from it, under light illumination, it is observed that the lifetimes of photogenerated electrons in Cd_1−x_S are decreased from 2.95 to 0.44 ns (Au_SA_/Cd_1−x_S) and 2.22 (Au_NC_/Cd_1−x_S) (cf. Supplementary Fig. 28). It could be concluded that, compared with Au nanoclusters, the stronger interaction between single Au atoms and Cd vacancies significantly accelerates electron transfer to the surface to enhance the surface electron density under light illumination. Meanwhile, the intensity of transient photocurrent response is significantly increased with the introduction of Au (cf. Fig. 7a). Au_SA_/Cd_1−x_S sample reveals the highest intensity compared with Au_NC_/Cd_1−x_S and Cd_1−x_S. It is suggested that single Au atoms offer a significantly higher enhancement in the surface electron density of Cd_1−x_S compared with Au clusters. This is further identified by the surface photovoltage (SPV) in Fig. 7b. It should be noted that two peaks are appearing in the SPV spectra of Cd_1−x_S. The peak at c.a. 400 nm corresponds to the electrons transferring from the top of valence band to the bottom of conduction band. The other one at c.a. 500 nm is assigned to the defect level introduced by Cd vacancy on the surface which is observed in the band structure (cf. Supplementary Fig. 29). Interestingly, intensities of the two peaks increase after anchoring Au nanostructures, especially the single Au atoms. It is suggested that compared with Au nanoclusters, the introduction of single Au atoms significantly promotes the enhancement of electron density on the Cd_1−x_S surface as well as the Cd vacancies under light illumination. Meanwhile, a similar trend is observed in transient photocurrent response and SPV spectra of Au/CdS_1−x_ system (cf. Supplementary Fig. 30).

As presented in Fig. 7c and Supplementary Fig. 31, the processes of CO_2_ reduction on Au_SA_/Cd_1−x_S and Au_NC_/Cd_1−x_S are further simulated by DFT calculations. Most of the energy barriers on Au_SA_/Cd_1−x_S are lower than that of Au_NC_/Cd_1−x_S, indicating that single Au atoms greatly facilitate CO_2_ reduction compared with Au nanoclusters. More interestingly, the energy barrier of the first step (CO_2_+H→COOH) on Au_SA_/Cd_1−x_S is remarkably decreased by 91.1% relative to that over Au_NC_/Cd_1−x_S. This suggests that the accumulated electrons in Cd vacancies on Au_SA_/Cd_1−x_S not only promote the activation of CO_2_ molecule, but also facilitate its further transformations. It is identified that there is no significant absorption band assigned to COOH* observed among in situ DRIFTS test over Au_SA_/Cd_1−x_S. Furthermore, extra electron is introduced to create an electron-rich condition in the process of CO_2_ transition into COOH in Fig. 7d^53^. Interestingly, the energy barrier on Au_SA_/Cd_1−x_S presents a larger decrease (41%) compared with that on Au_NC_/Cd_1−x_S (34%). It is suggested that, compared with Au_NC_/Cd_1−x_S, the enhancement of surface electron density much more facilitates the occurrence of CO_2_ reduction catalyzed by Au_SA_/Cd_1−x_S.

In summary, we have designed and fabricated a series of Au/CdS photocatalysts. On the one hand, the types of vacancies highly affect the direction of electron transfer. That is the electrons will transfer from CdS_1−x_ to Au in CdS_1−x_ system. Quite different from it, the direction of electron transfer has been reversed in Cd_1−x_S system, resulting in the accumulation of electrons in Cd vacancies. Hence, the adsorption of CO_2_ is enabled in vacancy sites instead of Au sites. More importantly, there is a stable electron-donation channel forming between CO_2_ and Cd vacancies, accelerating the transfer of accumulated electrons to CO_2_ for its effective activation. On the other hand, under light illumination, when Au nanostructures change from Au nanoclusters to single Au atoms, the surface electron density is significantly increased and promotes much more electrons accumulating on the surface as well as the Cd vacancies, which is beneficial for the subsequent reaction. As a result, the CO and CH_4_ generation rates of Au_SA_/Cd_1−x_S manifest a remarkable 160-fold and 113-fold enhancement compared with Cd_1−x_S as well as the H_2_ generation rate is promoted from 0 to 7.9 μmol g^−1^ h^−1^.

## Methods

### Synthesis of Cd_1−x_S, CdS_1−x_ and CdS

The Cd_1−x_S and CdS_1−x_ were synthesized via a modified hydrothermal method^54^. For Cd_1−x_S, the synthesis process as follows: first, CdCl_2_·5H_2_O (4 mM) was dissolved in 20 mL aqueous solution (pH = 1, marked as solution A). Meanwhile, another 20 mL aqueous Na_2_S·9H_2_O (8 mM) solution was prepared, marked as solution B. Then, solution B was added dropwise into solution A under continuous stirring. The mixed solution was transferred into a Teflon-lined stainless steel autoclave (inner volume: 50 mL) and held at 140 °C for 18 h in an oven. After cooling to room temperature naturally, the product was centrifuged and washed with water and ethyl alcohol several times. Finally, the obtained product was dried overnight at 60 °C under vacuum condition. The synthesis process of CdS_1−x_ and CdS were the same as that of Cd_1−x_S except that 1 and 4 mM of Na_2_S^.^9H_2_O were added in solution B.

### Synthesis of Au/Cd_1−x_S and Au/CdS_1−x_

The Au/Cd_1−x_S and Au/CdS_1−x_ were synthesized by the impregnation method. For 1%Au/Cd_1−x_S, 100 mg of Cd_1−x_S was dispersed in 30 mL water and adding 0.2 mL HAuCl_4_ solution (5 mg/mL). Then, the mixed solution was stirred at 70 °C for 6 h. The product was centrifuged and washed with water and ethyl alcohol several times and dried overnight at 60 °C under vacuum condition. The synthesis procedure of 2%Au/Cd_1−x_S was the same as that of 1%Au/Cd_1−x_S, except that 0.4 mL of HAuCl_4_ solution was used in the experiment. Meanwhile, Au/CdS_1−x_ samples were synthesized by the same method using CdS_1−x_ instead of Cd_1−x_S.

### Photocatalytic performance measurement

Photocatalytic CO_2_ reduction performance test was carried out in a closed gas system (volume: 276 mL) at atmospheric pressure and ambient temperature. First, 30 mg of photocatalyst was dispersed with H_2_O in a petri dish (ø = 6 cm) and dried at 70 °C under vacuum condition. Then, petri dish was put on a stainless steel breaker with 1 mL of deionized water in the reactor, which was filled with the CO_2_ (concentration: 1000 ppm; carrier gas: Ar). A 300 W Xe lamp was used as the UV-vis light source (intensity: 600 mW/cm^2^). Every 2 h, 1 mL of gas was taken from the reactor for qualitative analysis by gas chromatography (GC7900, Tianmei Analytical Instrument Co., Ltd., China). CO and CH_4_ were detected by a flame ionization detector (FID) and a thermal conductivity detector (TCD) was used for detecting H_2_. The isotope test was carried out by using ^13^CO_2_ as the carbon source with the same reaction set as mentioned above and the gas products were analyzed by mass spectrometry (OmniStar^TM^, Pfeiffer Vacuum, Germany).

### In situ DRIFTS investigation

In situ DRIFTS measurements were performed on a TENSOR II FT-IR spectrometer (Bruker, Germany). The spectrometer was equipped with an in situ diffuse reflectance cell (Harrick) and a high-temperature reaction chamber (HVC). A mixture of ^12^CO_2_ (20% CO_2_/Ar) and H_2_O vapors (humidity: 5%Rh) with a flow rate of 17 mL min^−1^ was introduced into the Harrick cell. In case of Au_SA_/Cd_1−x_S and Au_SA_/CdS_1−x_ samples, ^13^CO_2_ (20% ^13^CO_2_/Ar) was used instead of ^12^CO_2_ to perform the isotope test. For CO probe test, a mixture of CO (10% CO/Ar) and H_2_O vapors (humidity: 5%Rh) with a flow rate of 17 mL min^−1^ was introduced into the Harrick cell. Before the measurement, the samples were pretreated at 100 °C for 10 min under Ar atmosphere.

## Supplementary information

Supplementary Information

## Data Availability

The data that support the findings of this study are available from the corresponding author upon reasonable request.

## Code availability

The code to reproduce the results is available from the corresponding author upon reasonable request.
