# Supplementary material for: Modulating electron density of vacancy site by single Au atom for effective CO2 photoreduction
Source: Nat Commun. 2021 Mar 15;12:1675. doi: 10.1038/s41467-021-21925-7 (PMC7960986; doi:10.1038/s41467-021-21925-7)
Supplement: Supplementary file 1 — Supplementary Information [file 41467_2021_21925_MOESM1_ESM.pdf]

**Supplementary Information (SI)**

# Modulating electron density of vacancy site by single Au atom for effective $CO_2$ photoreduction

Cao et al.

# Table of contents

1. Selection of loading metal ........ S4

2. TEM and HRTEM images of $Cd_{1-x}S$ and Au/$Cd_{1-x}S$........ S5

3. EPR pattern of $Cd_{1-x}S$ ........ S6

4. Size distributions of Au nanoclusters and EDS mapping images of 2%Au/$Cd_{1-x}S$ ........ S7

5. EPR patterns of $Cd_{1-x}S$ and $Au_{SA}/Cd_{1-x}S$ ........ S8

6. XRD patterns of $CdS_{1-x}$ system........ S9

7. EPR pattern of $CdS_{1-x}$ ........ S10

8. Cd K edge XANES and EXAFS spectra of $CdS_{1-x}$ and CdS ........ S11

9. Cd K edge XANES and EXAFS spectra of 1%Au/$CdS_{1-x}$ and $CdS_{1-x}$ ........ S12

10. Au $L_3$ edge XANES and EXAFS spectra of 1%Au/$CdS_{1-x}$........ S13

11. Photoreduction $CO_2$ performance of $Cd_{1-x}S$ and $CdS_{1-x}$ systems........ S14

12. Isotope experiment........ S15

13. XRD patterns of fresh and used $Au_{SA}/Cd_{1-x}S$ samples ........ S16

14. TEM and HRTEM images of fresh and used $Au_{SA}/Cd_{1-x}S$ samples........ S17

15. UV-vis spectra ........ S18

16. In situ DRIFTS spectra of $CdS_{1-x}$ system........ S19

17. $CO_2$ adorption configurations of $Au_{NC}/CdS_{1-x}$........ S20

18. $CO_2$ adorption configurations of $Au_{NC}/Cd_{1-x}S$........ S21

19. BET of $Cd_{1-x}S$ system........ S22

20. $CO_2$ adsorption isotherms........ S23

21. In situ DRIFTS spectra of CO adsorption ........ S24

22. Electrostatic potential energy........ S25

23. Charge population........ S26

24. XPS spectra of $Cd_{1-x}S$ system ........ S27

25. XPS spectra of $CdS_{1-x}$ system ........ S28

26. DOS of $Au_{SA}/Cd_{1-x}S$ and $Au_{NC}/Cd_{1-x}S$........ S29

27. PDOS of Au 5d orbits in $Au_{SA}$, $Au_{SA}/Cd_{1-x}S$, $Au_{NC}$ and $Au_{NC}/Cd_{1-x}S$ ........ S30

28. TR-PL spectra of $Cd_{1-x}S$, $Au_{SA}/Cd_{1-x}S$ and $Au_{NC}/Cd_{1-x}S$ samples........ S31

29. Band structure of CdS and $Cd_{1-x}S$ system........ S32

30. Transient photocurrent response and SPV spectra of $CdS_{1-x}$ and Au/$CdS_{1-x}$............................S33

31. Global reaction profiles for $CO_2$ reduction in $Cd_{1-x}S$ system ....................................................S34

32. Elemental composition ..........................................................................................................S35

33. Au coordination environments determined from the analysis of EXAFS spectra.......................S36

34. Formation energy of single Au atoms loaded $Cd_{1-x}S$ surface...................................................S37

35. Recent improvement of photocatalytic conversion of $CO_2$ ..................................................S38

36. Adsorption energy of $CO_2$..........................................................................................................S39

37. Experiments...............................................................................................................................S40

38. Calculation method and model..................................................................................................S42

39. References ..................................................................................................................................S43

## 1. Selection of loading metal

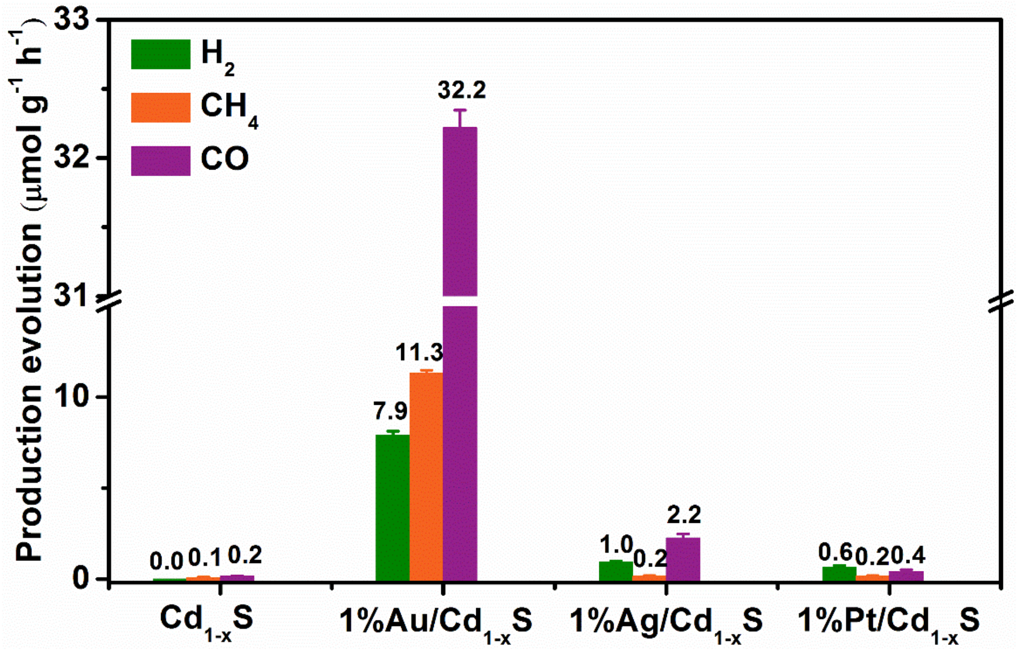


**Supplementary Fig. 1** Average yield in the photocatalytic conversion of $CO_2$ with $H_2O$ under UV-visible light over pristine $Cd_{1-x}S$, 1%Au/$Cd_{1-x}S$, 1%Ag/$Cd_{1-x}S$ and 1%Pt/$Cd_{1-x}S$ samples. The error bar was drawn based on the caluculated standard error of two parallel tests.

## 2. TEM and HRTEM images of $Cd_{1-x}S$ and $Au/Cd_{1-x}S$

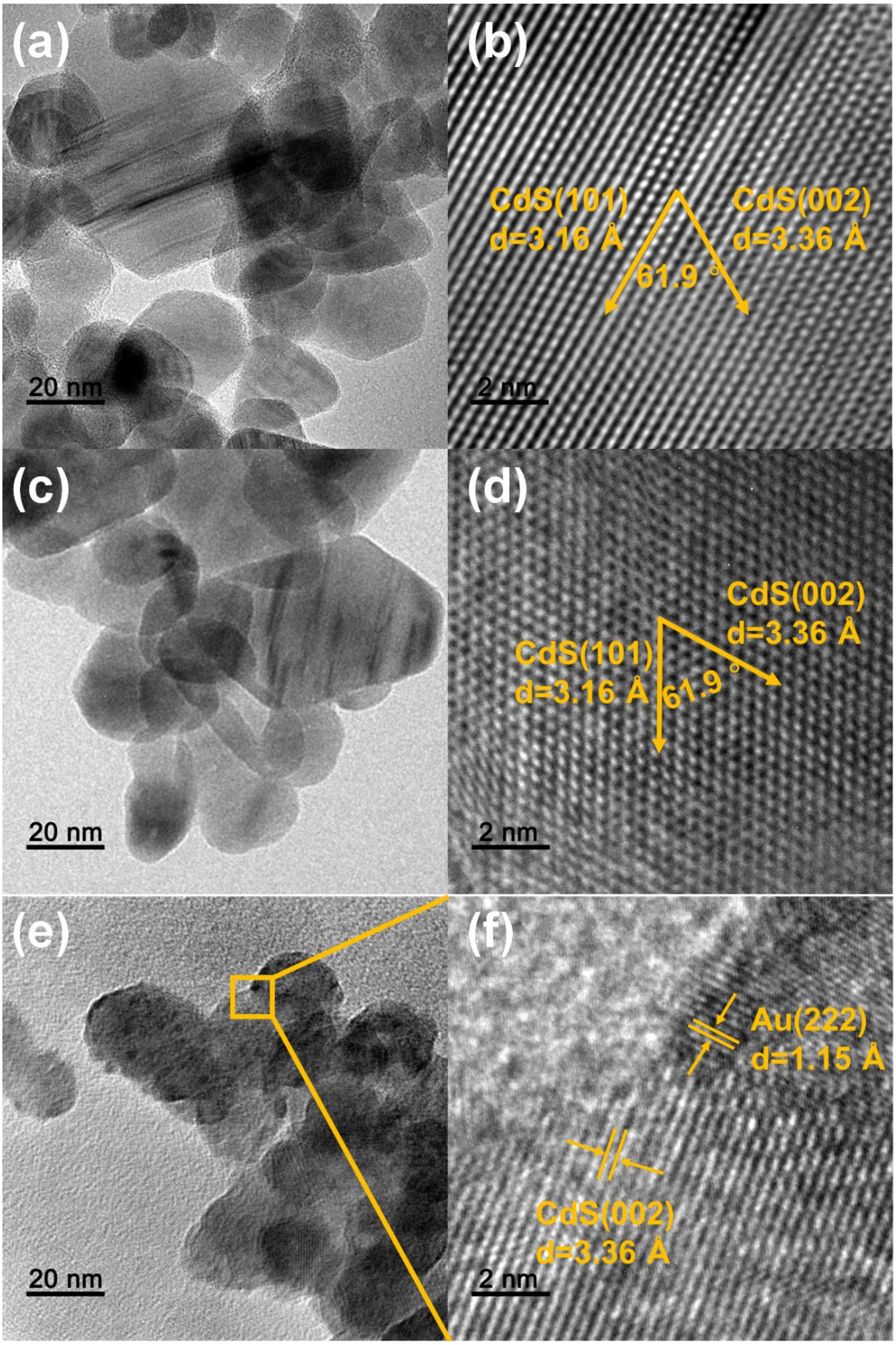


**Supplementary Fig. 2** TEM and HRTEM images of $Cd_{1-x}S$ (a, b), 1%$Au/Cd_{1-x}S$ (c, d) and 2%$Au/Cd_{1-x}S$ (e, f) samples.

**3. EPR pattern of $Cd_{1-x}S$**

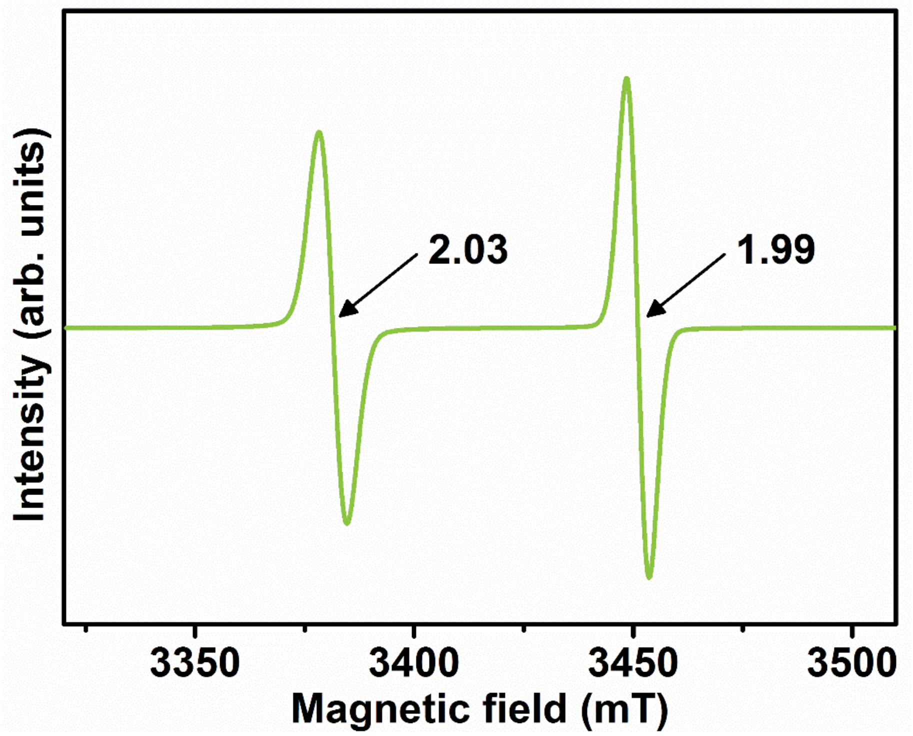


**Supplementary Fig. 3** EPR pattern of $Cd_{1-x}S$ sample.

## 4. Size distributions of Au nanoclusters and EDS mapping images of 2%Au/$Cd_{1-x}S$

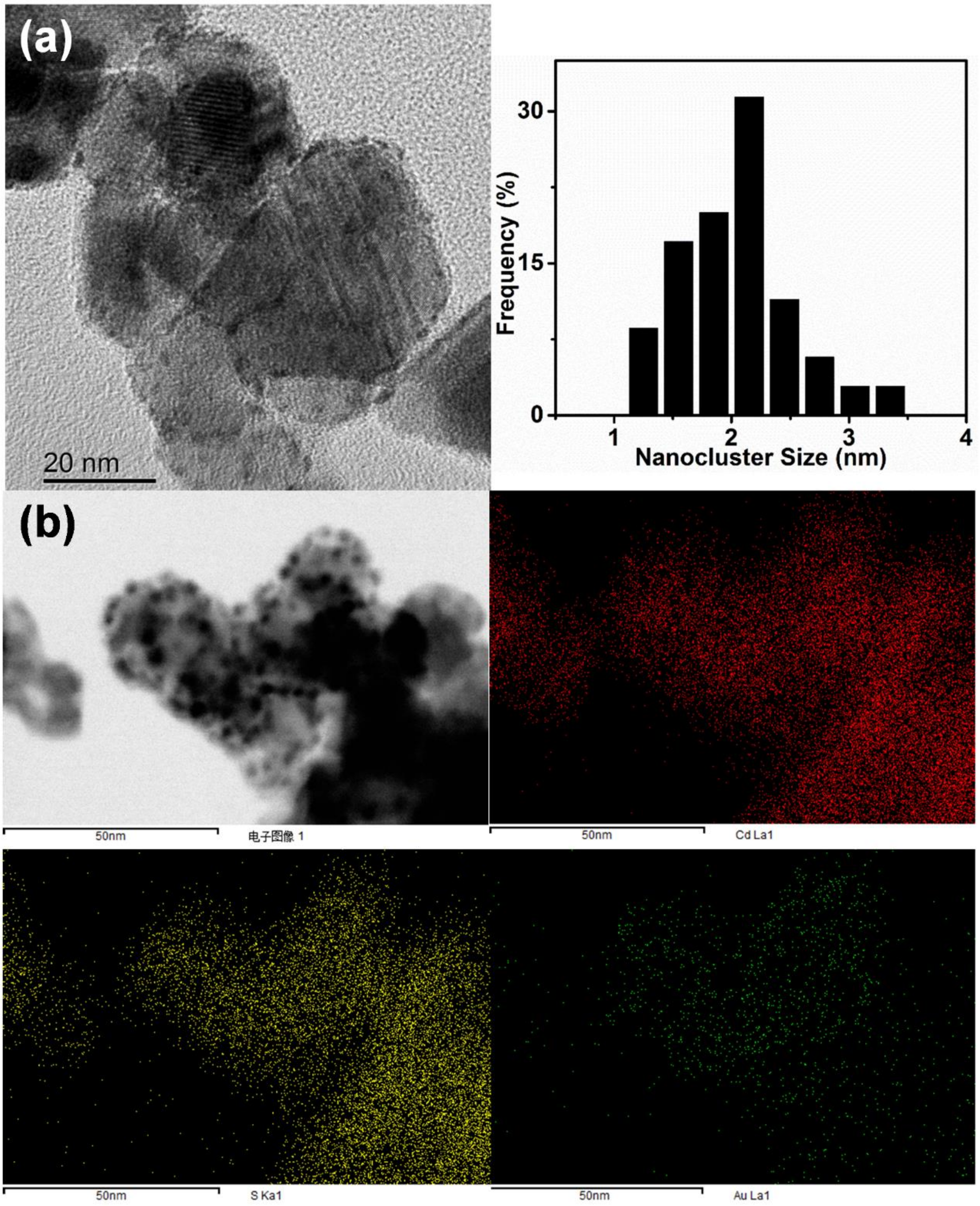


**Supplementary Fig. 4** TEM image with the size distributions of Au nanoclusters (a) and EDS Mapping (b) images of 2%Au/$Cd_{1-x}S$ sample.

## 5. EPR patterns of $Cd_{1-x}S$ and $Au_{SA}/Cd_{1-x}S$

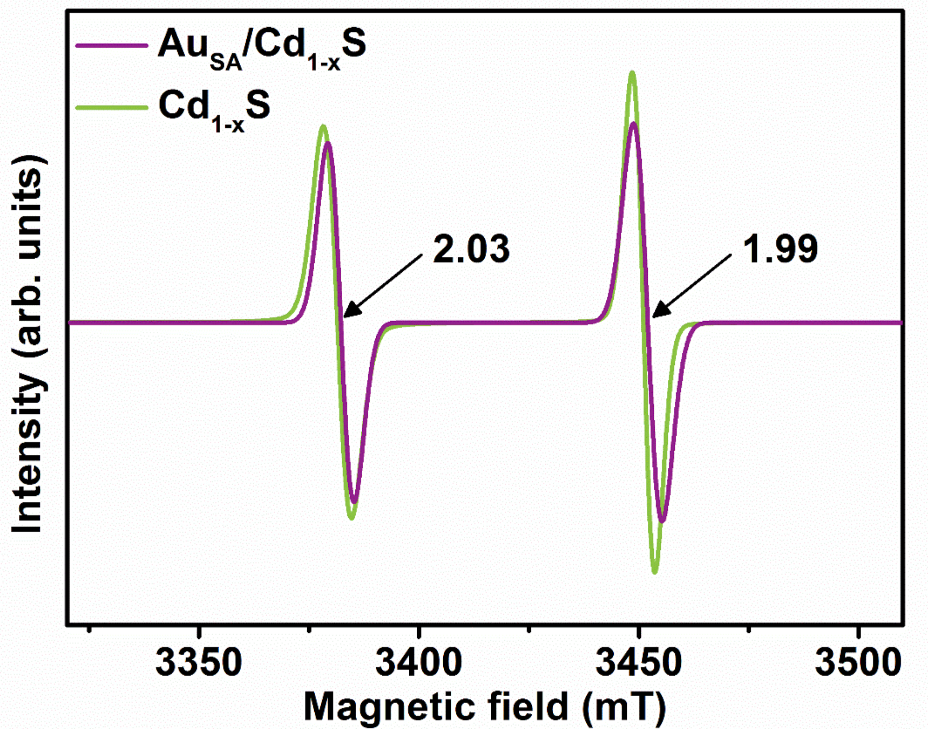


**Supplementary Fig. 5** EPR patterns of $Cd_{1-x}S$ and $Au_{SA}/Cd_{1-x}S$ samples.

## 6. XRD patterns of $CdS_{1-x}$ system

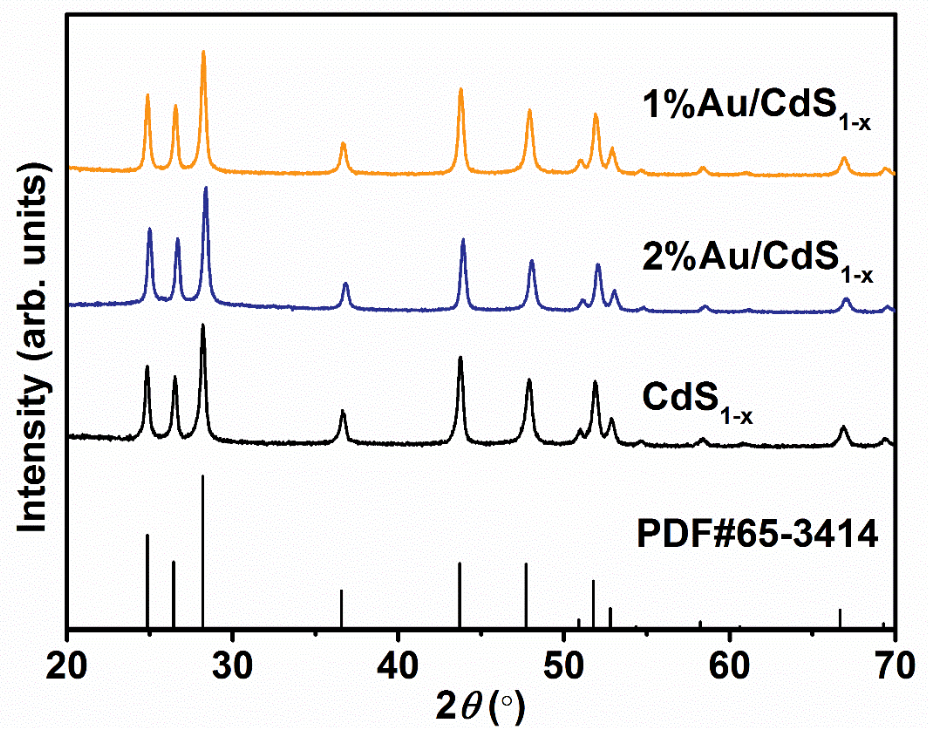


**Supplementary Fig. 6** XRD patterns of $Cd_{1-x}S$, 1%Au/$CdS_{1-x}$ and 2%Au/$CdS_{1-x}$ samples.

**7. EPR pattern of $CdS_{1-x}$**

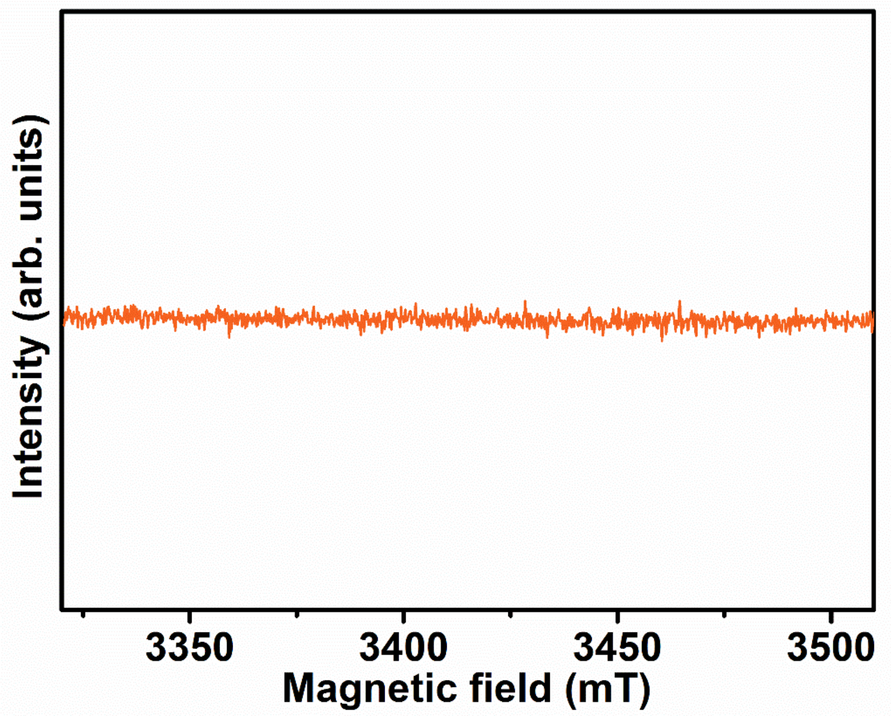


**Supplementary Fig. 7** EPR pattern of $CdS_{1-x}$ sample.

## 8. Cd K edge XANES and EXAFS spectra of $CdS_{1-x}$ and CdS

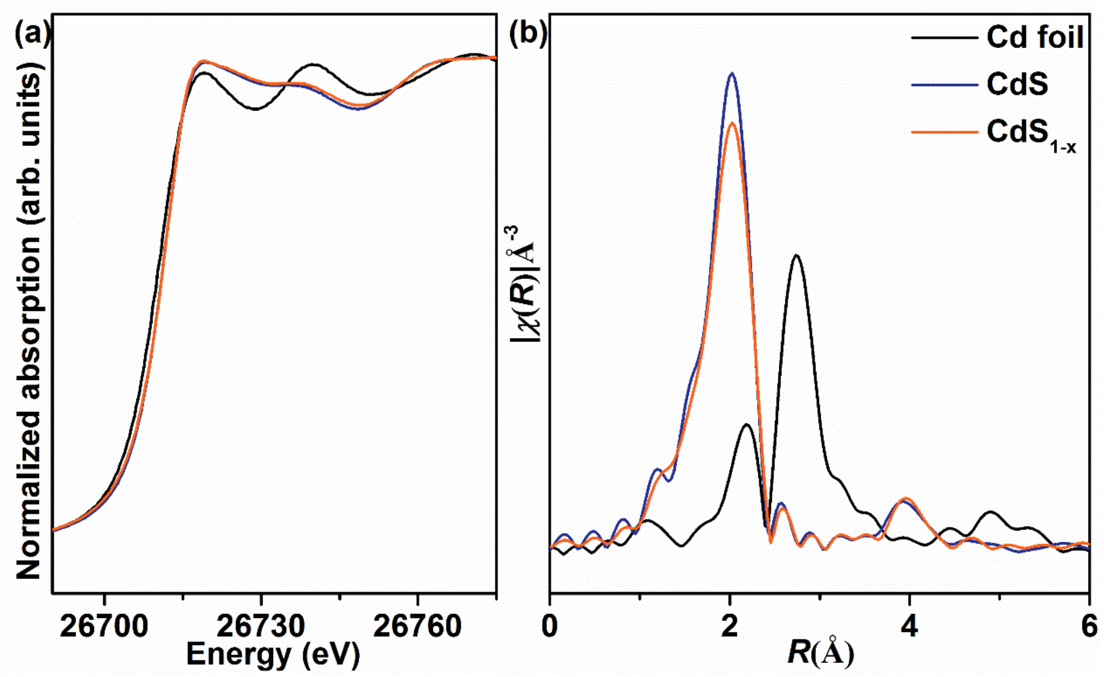


**Supplementary Fig. 8** XAFS analysis of CdS and $CdS_{1-x}$: Cd K XANES spectra of the sample and the reference foil (a); Corresponding $k^1$-weighted Fourier transform (FT) EXAFS spectra (b).

9. Cd K edge XANES and EXAFS spectra of 1%Au/$CdS_{1-x}$ and $CdS_{1-x}$

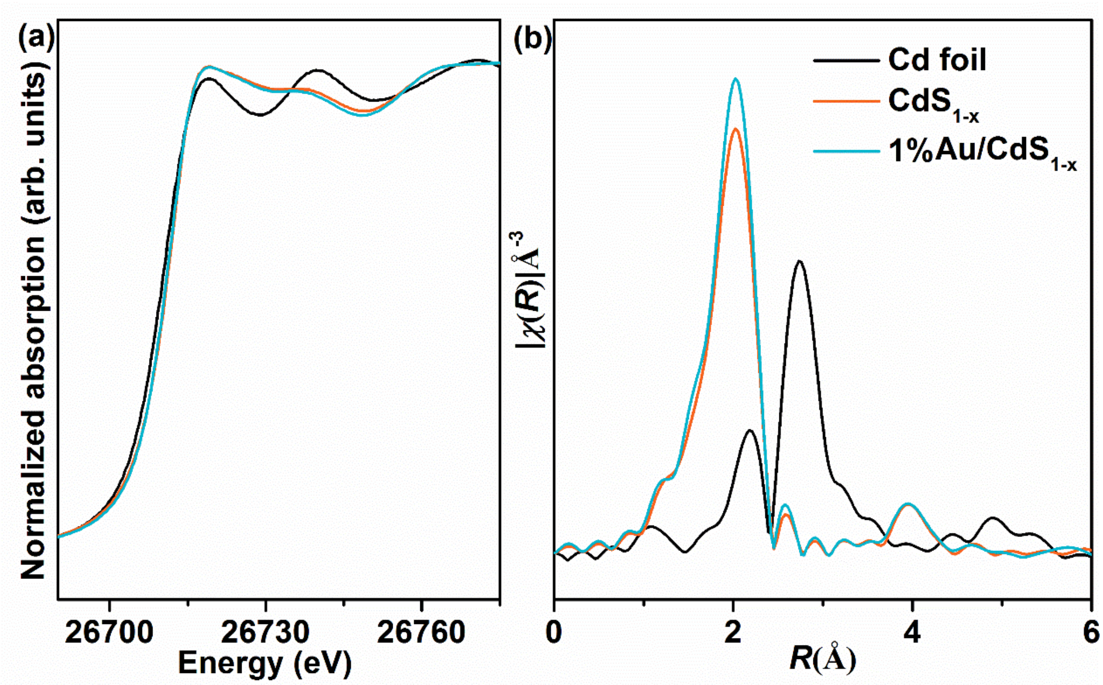


**Supplementary Fig. 9** XAFS analysis of 1%Au/$CdS_{1-x}$ and $CdS_{1-x}$: Cd K XANES spectra of the sample and the reference foil (a); Corresponding $k^1$-weighted Fourier transform (FT) EXAFS spectra (b).

**10. Au $L_3$ edge XANES and EXAFS spectra of 1%Au/$CdS_{1-x}$**

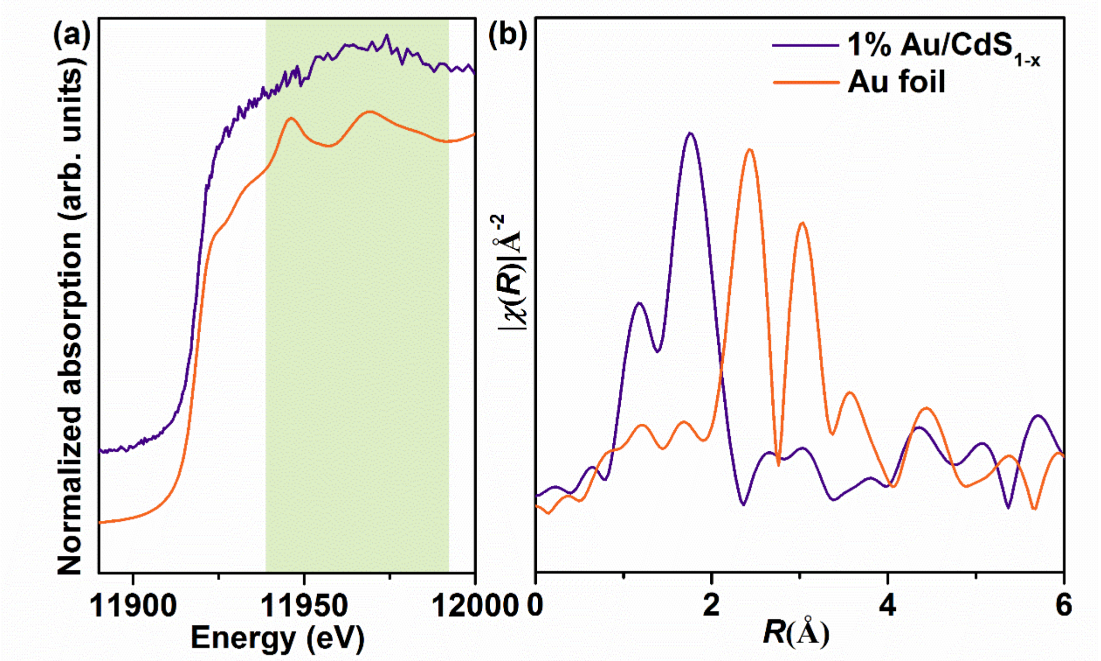


**Supplementary Fig. 10** XAFS analysis of 1%Au/$CdS_{1-x}$ and $CdS_{1-x}$: Au $L_3$ XANES spectra of the sample and the reference foil (a); Corresponding $k^1$-weighted Fourier transform (FT) EXAFS spectra (b).

## 11. Photoreduction $CO_2$ performance of $Cd_{1-x}S$ and $CdS_{1-x}$ systems

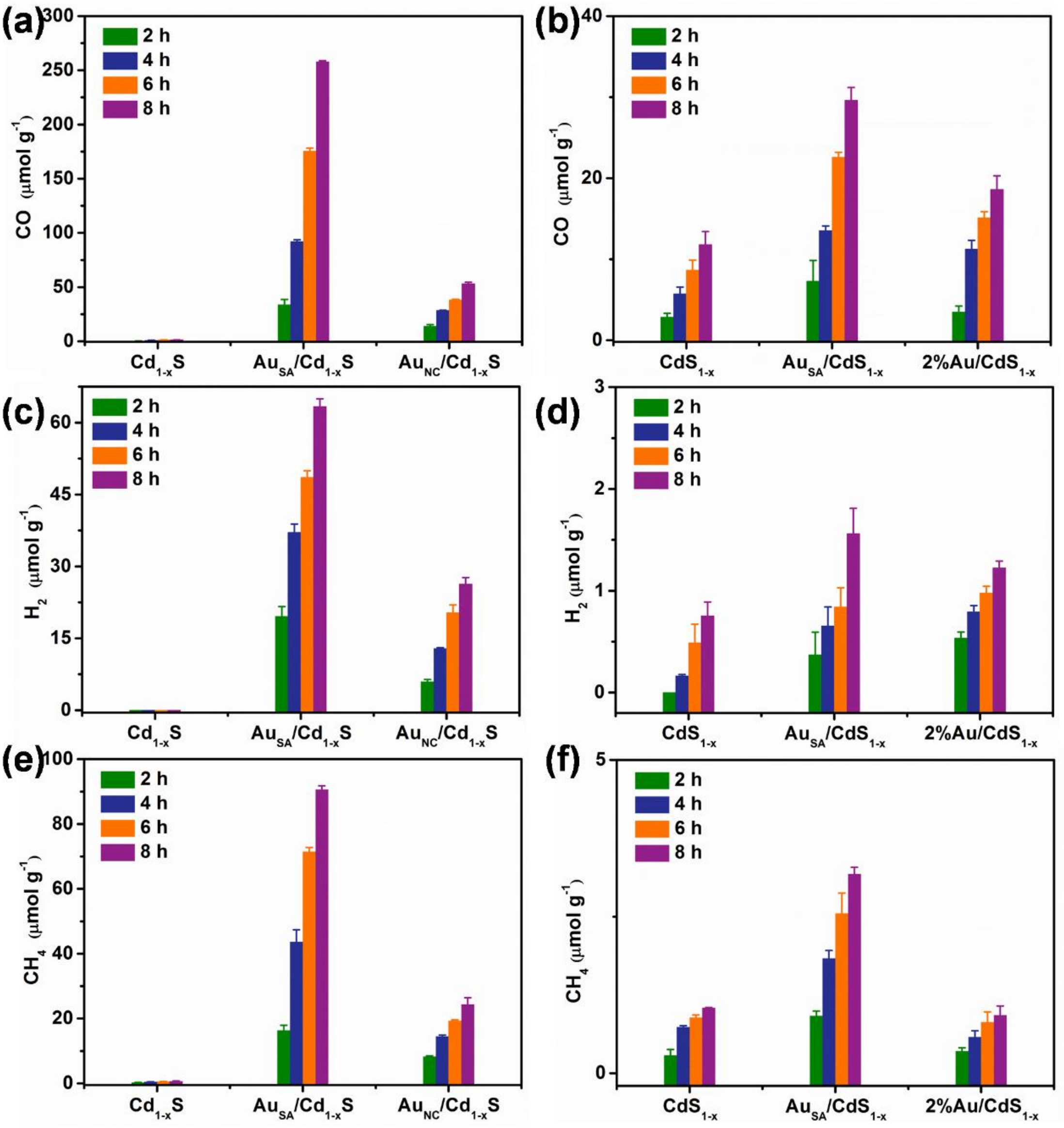


**Supplementary Fig. 11** Production of CO (a-b), $H_2$ (c-d) and $CH_4$ (e-f) over $Cd_{1-x}S$, $Au_{SA}/Cd_{1-x}S$, $Au_{NC}/Cd_{1-x}S$, $CdS_{1-x}$, $Au_{SA}/CdS_{1-x}$ and 2%Au/$CdS_{1-x}$ samples in the photocatalytic conversion of $CO_2$ with $H_2O$ in the presence of water vapor under UV-visible light. The error bar was drawn based on the caluculated standard error of two parallel tests.

## 12. Isotope experiment

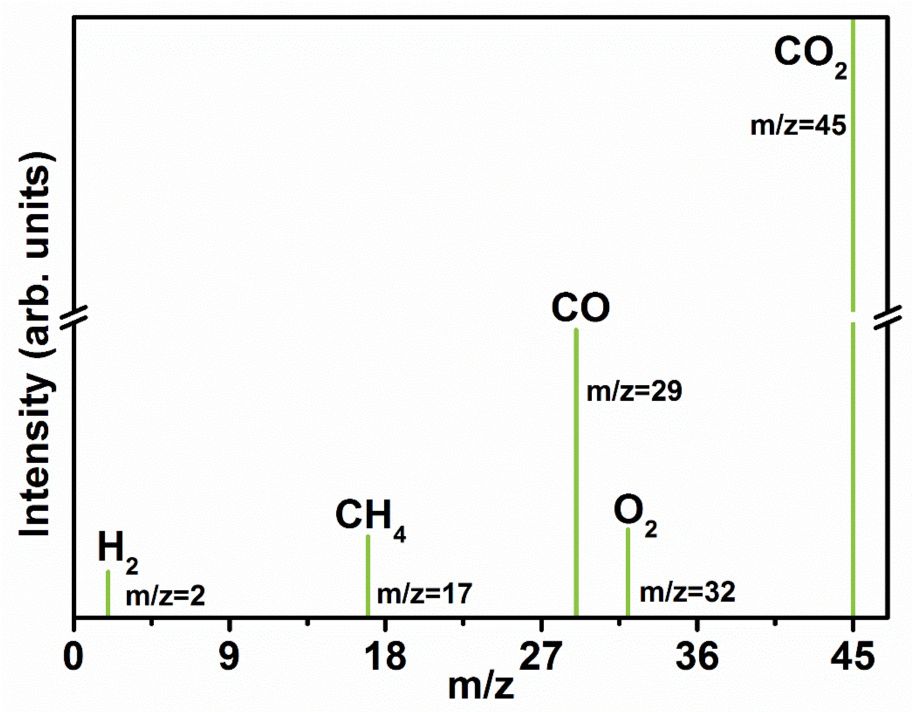


**Supplementary Fig. 12** Isotope experiment over $Au_{SA}/Cd_{1-x}S$ sample in the photocatalytic conversion of $CO_2$ with $H_2O$ in the presence of water vapor under UV-visible light after 8 hours' reaction.

**13. XRD patterns of fresh and used $Au_{SA}/Cd_{1-x}S$ samples**

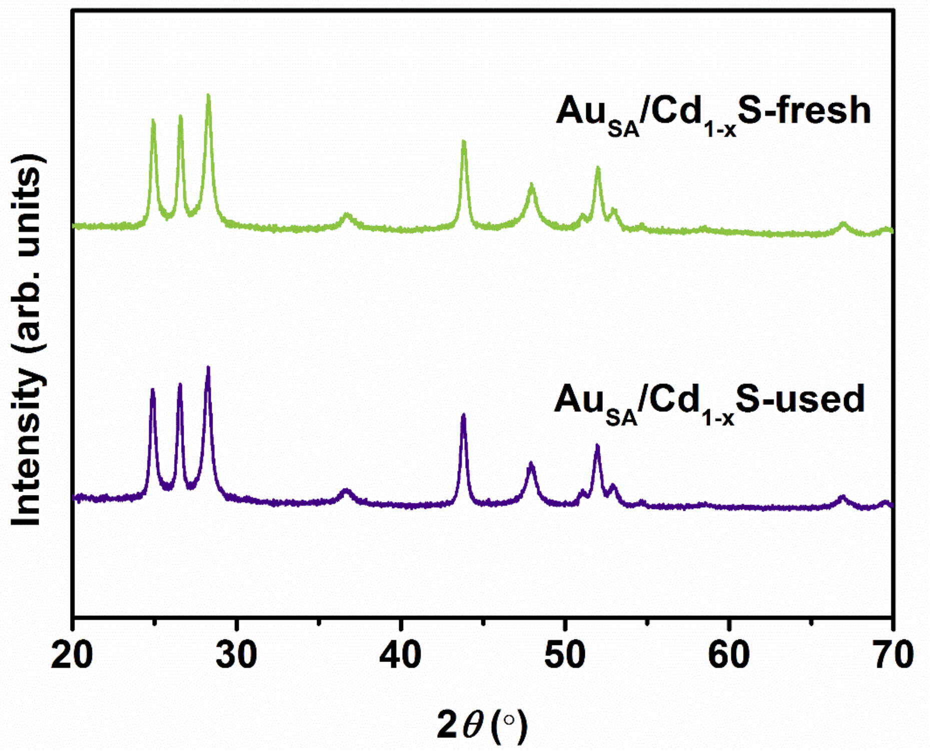


**Supplementary Fig. 13** XRD patterns of $Au_{SA}/Cd_{1-x}S$ sample before and after $CO_2$ photoreduction reaction for 8 hours.

**14. TEM and HRTEM images of fresh and used $Au_{SA}/Cd_{1-x}S$ samples**

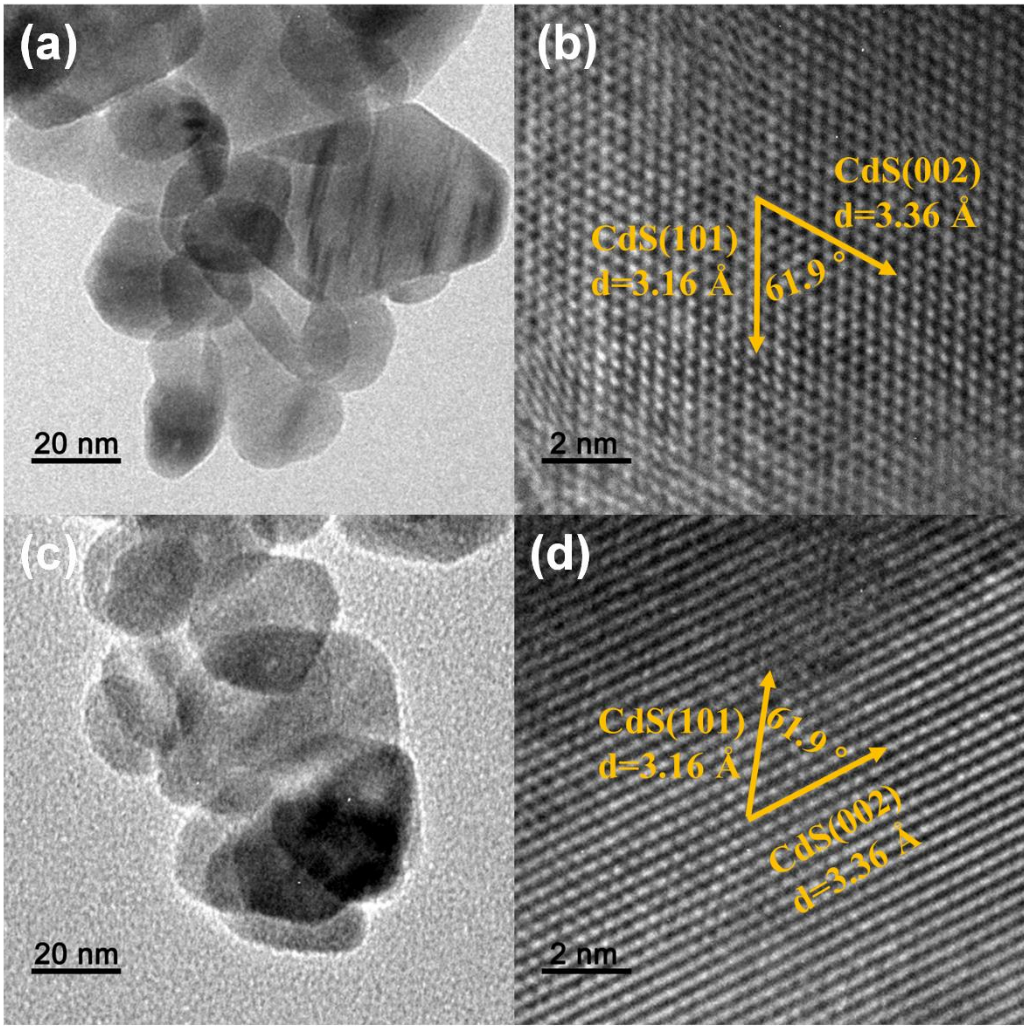


**Supplementary Fig. 14** TEM and HRTEM images of $Au_{SA}/Cd_{1-x}S$ sample before (a, b) and after (c, d) $CO_2$ photoreduction reaction for 8 hours.

## 15. UV-vis spectra

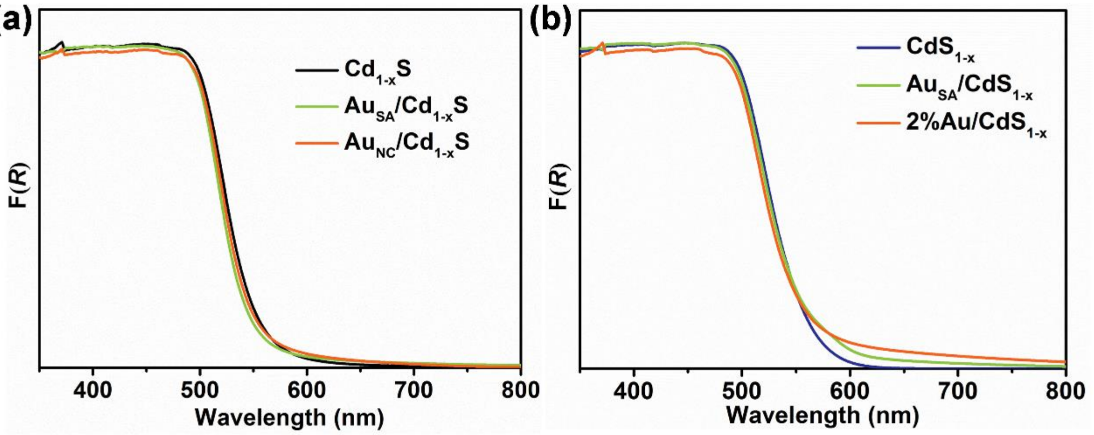


**Supplementary Fig. 15** UV-vis spectra of $Cd_{1-x}S$ (a) and $CdS_{1-x}$ (b) systems.

## 16. In situ DRIFTS spectra of $CdS_{1-x}$ system

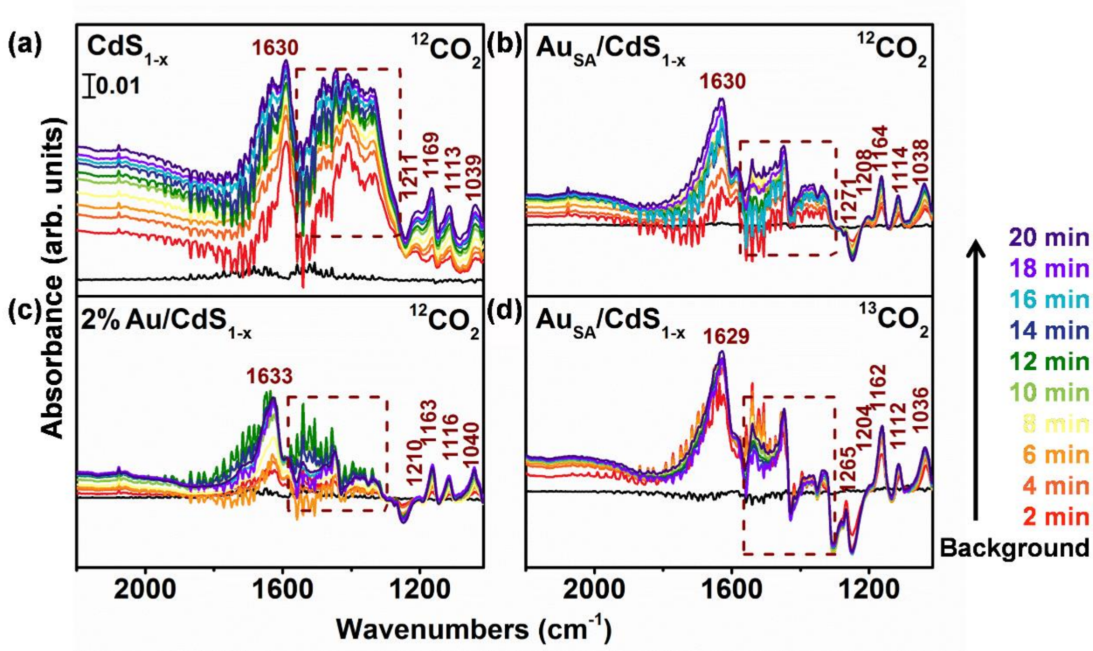


**Supplementary Fig. 16** In situ DRIFTS spectra of the $CO_2$ adsorption process over $CdS_{1-x}$ (a), $Au_{SA}/CdS_{1-x}$ (b and d) and 2%Au/$CdS_{1-x}$ (c) with $^{12}CO_2$ and $^{13}CO_2$.

## 17. $CO_2$ adorption configurations of $Au_{NC}/CdS_{1-x}$

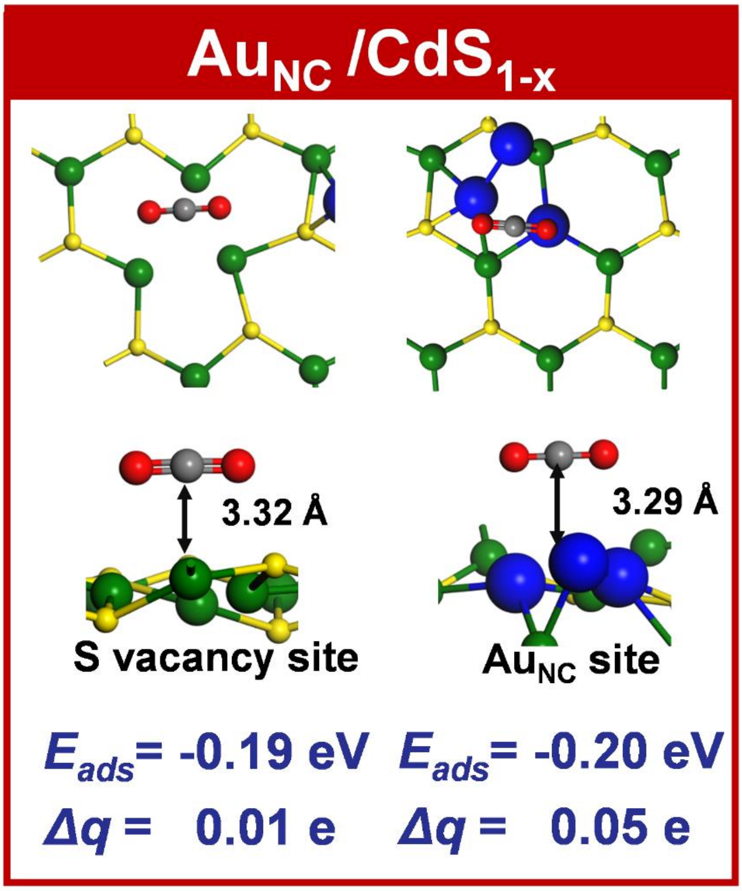


**Supplementary Fig. 17** Configurations of $CO_2$ adsorption on $Au_{NC}/CdS_{1-x}$ surface.

136 **18. $CO_2$ adorption configurations of $Au_{NC}/Cd_{1-x}S$**

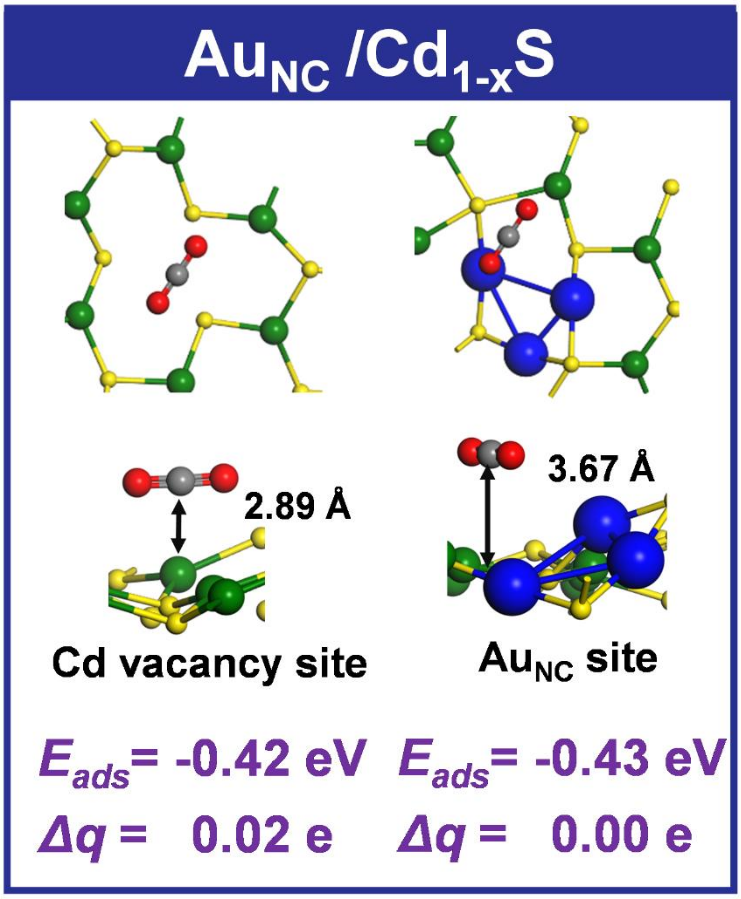


137

138 **Supplementary Fig. 18** Configurations of $CO_2$ adsorption on $Au_{NC}/Cd_{1-x}S$ surface.

139

**19. BET of $Cd_{1-x}S$ system**

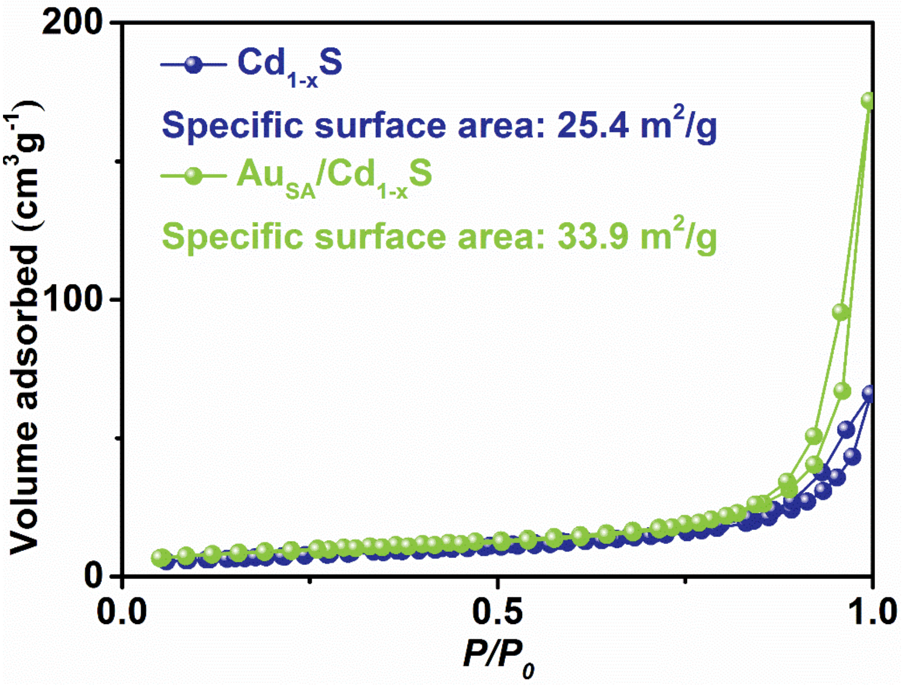


**Supplementary Fig. 19** BET of $Cd_{1-x}S$ and $Au_{SA}/Cd_{1-x}S$ samples.

**20. $CO_2$ adsorption isotherms**

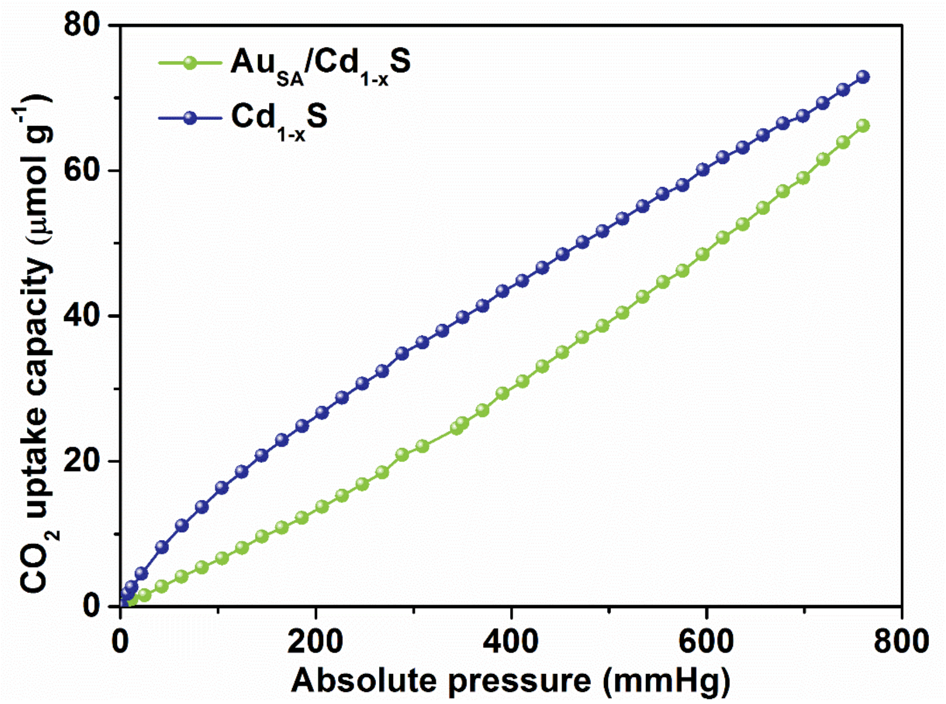


**Supplementary Fig. 20** $CO_2$ adsorption isotherms of $Cd_{1-x}S$ and $Au_{SA}/Cd_{1-x}S$ samples.

## 21. In situ DRIFTS spectra of CO adsorption

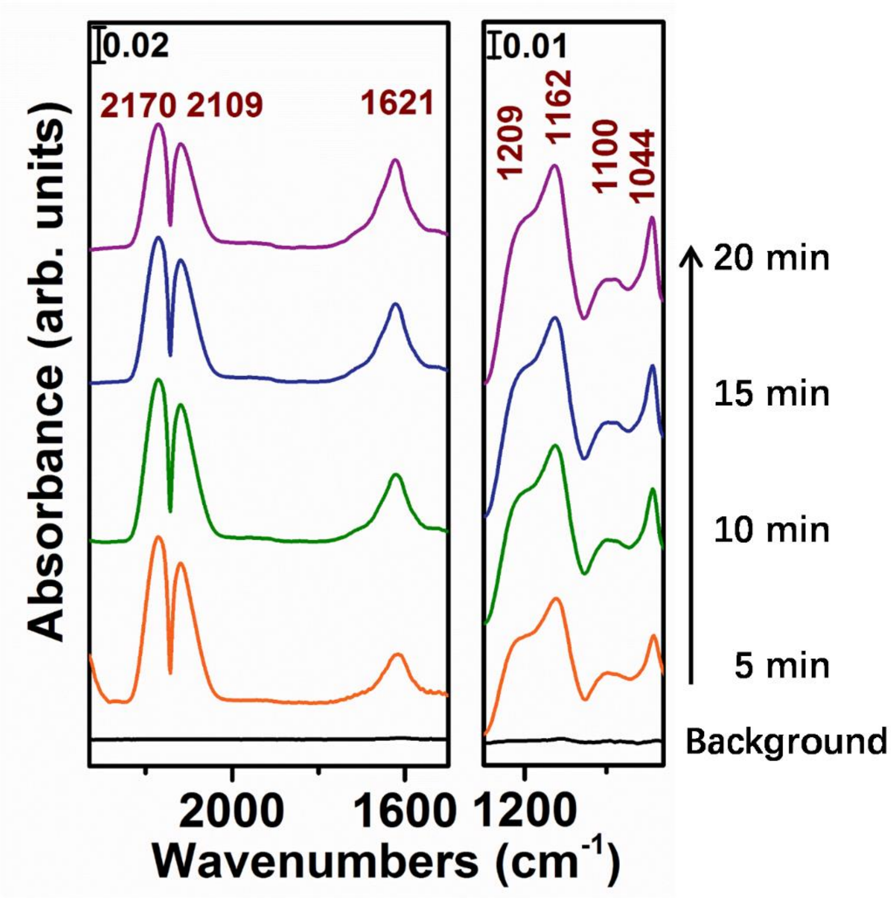


**Supplementary Fig. 21** In situ DRIFTS spectra of CO adsorption on $Au_{SA}/Cd_{1-x}S$.

## 22. Electrostatic potential energy

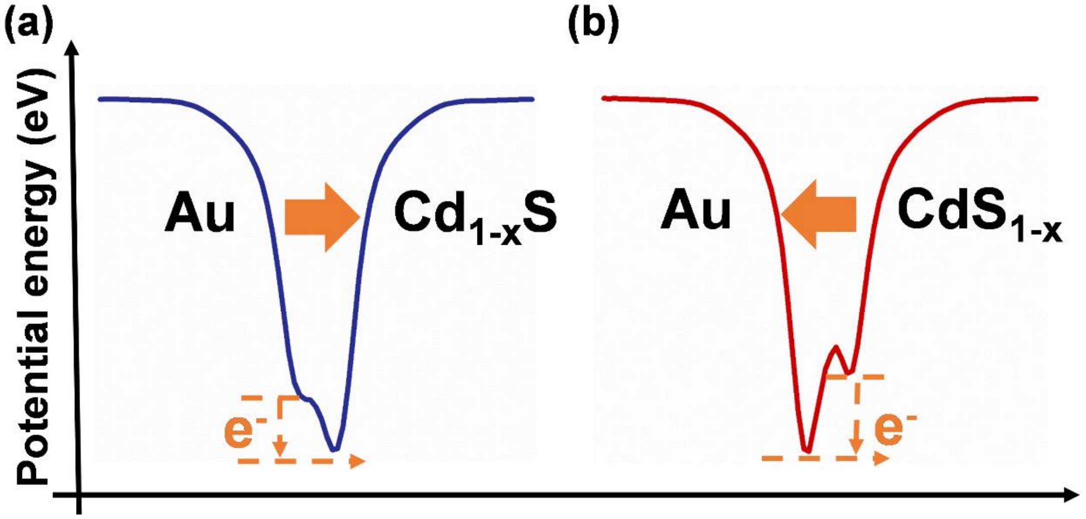


**Supplementary Fig. 22** Electrostatic potential energy of $Au_{SA}/Cd_{1-x}S$ (a) and $Au_{SA}/CdS_{1-x}$ (b).

## 23. Charge population

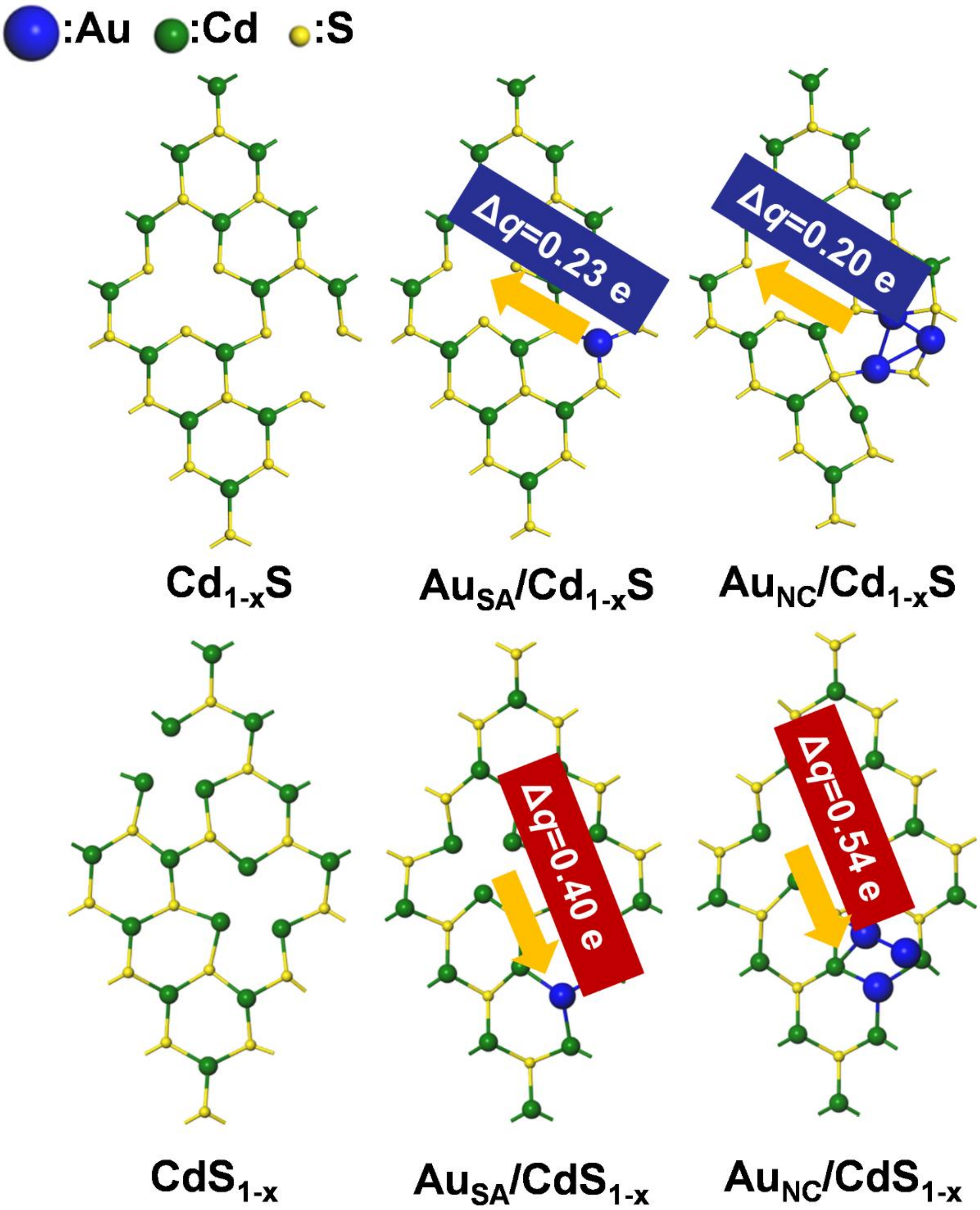


**Supplementary Fig. 23** Charge population of $Cd_{1-x}S$ and $CdS_{1-x}$ systems.

## 24. XPS spectra of $Cd_{1-x}S$ system

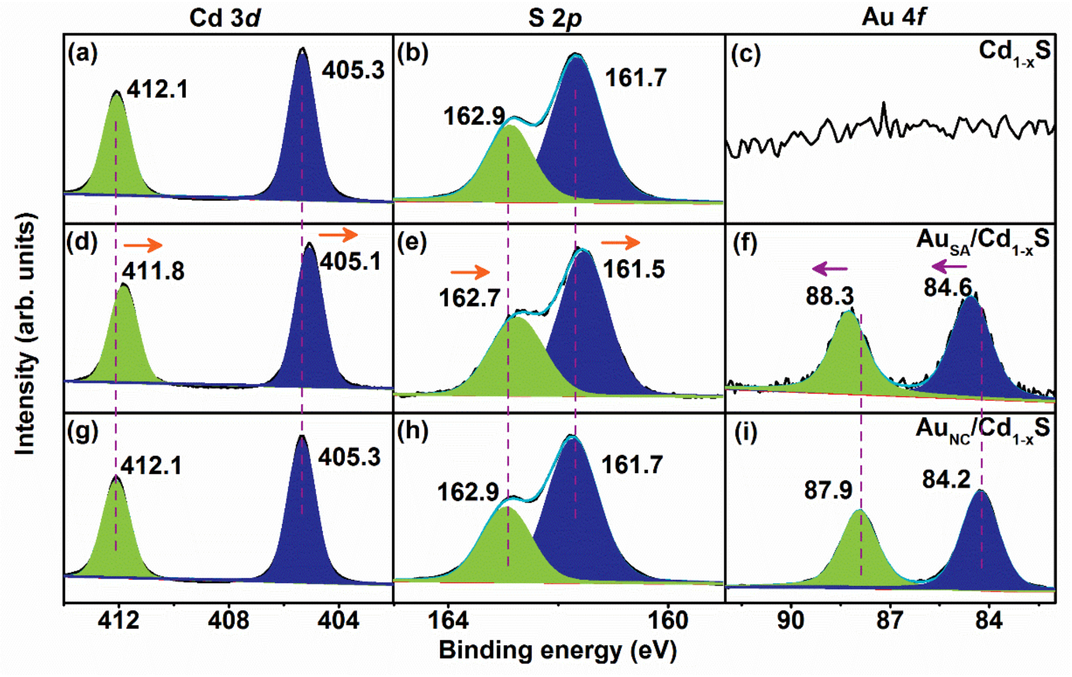


**Supplementary Fig. 24** XPS spectra of $Cd_{1-x}S$ and $Au_{SA}/Cd_{1-x}S$ and $Au_{NC}/Cd_{1-x}S$ samples: high-resolution spectra of Cd 3*d* (a, d, g), S 2*p* (b, e, h) and Au 4*f* (c, f, i).

## 25. XPS spectra of $CdS_{1-x}$ system

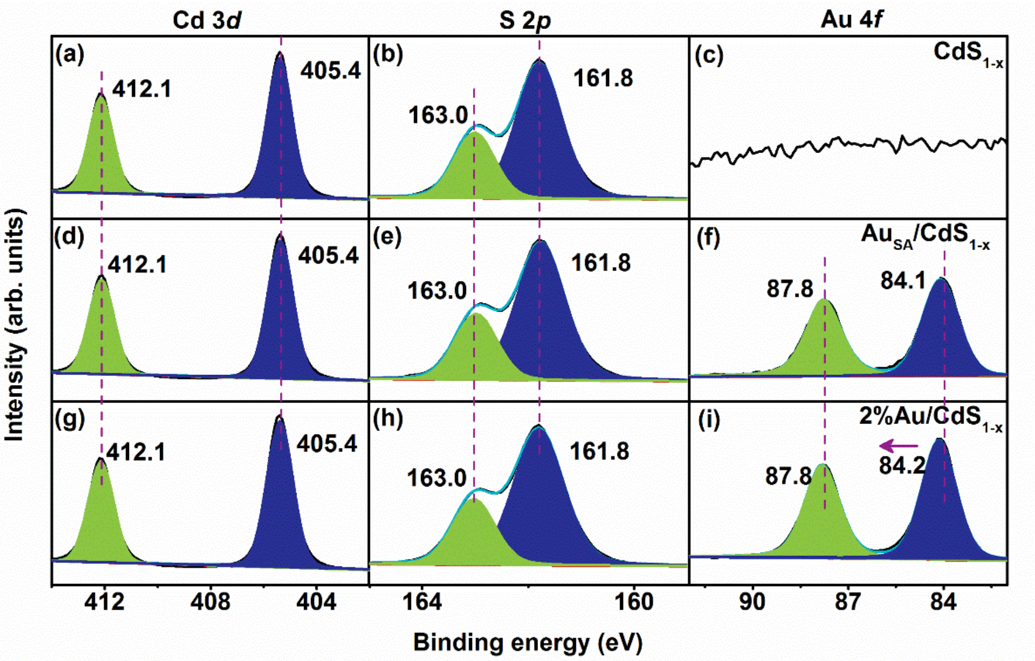


**Supplementary Fig. 25** XPS spectra of $CdS_{1-x}$ and $Au_{SA}/CdS_{1-x}$ and 2%Au/$CdS_{1-x}$ samples: high-resolution spectra of Cd 3*d* (a, d, g), S 2*p* (b, e, h) and Au 4*f* (c, f, i).

26. PDOS of $Au_{SA}/Cd_{1-x}S$ and $Au_{NC}/Cd_{1-x}S$

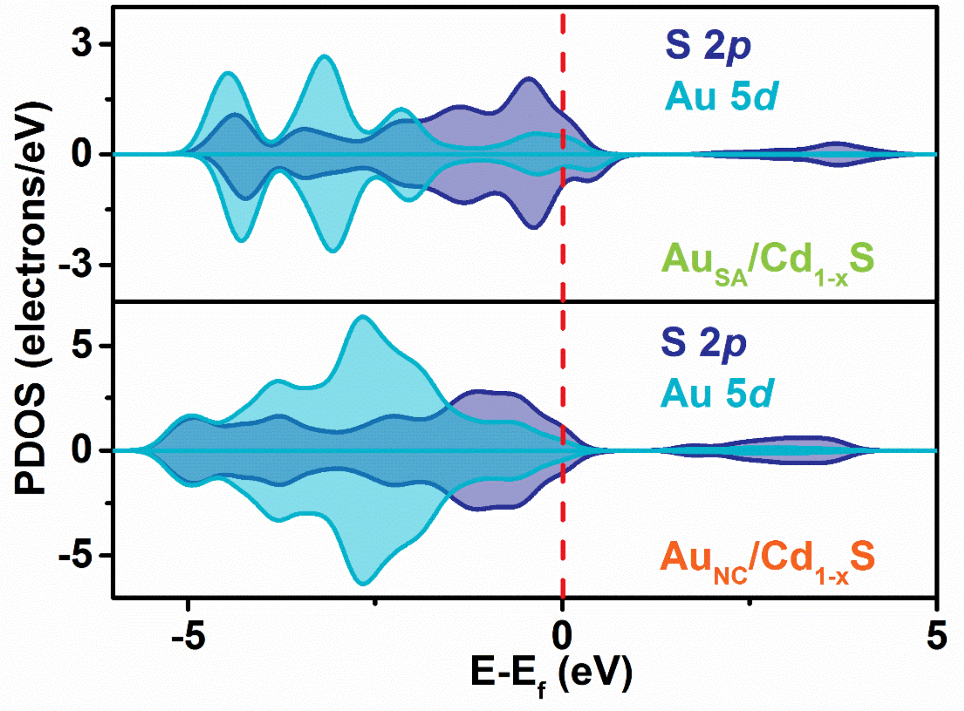


**Supplementary Fig. 26** PDOS of $Au_{SA}/Cd_{1-x}S$ and $Au_{NC}/Cd_{1-x}S$.

## 27. PDOS of Au 5d orbits in $Au_{SA}$, $Au_{SA}/Cd_{1-x}S$, $Au_{NC}$ and $Au_{NC}/Cd_{1-x}S$

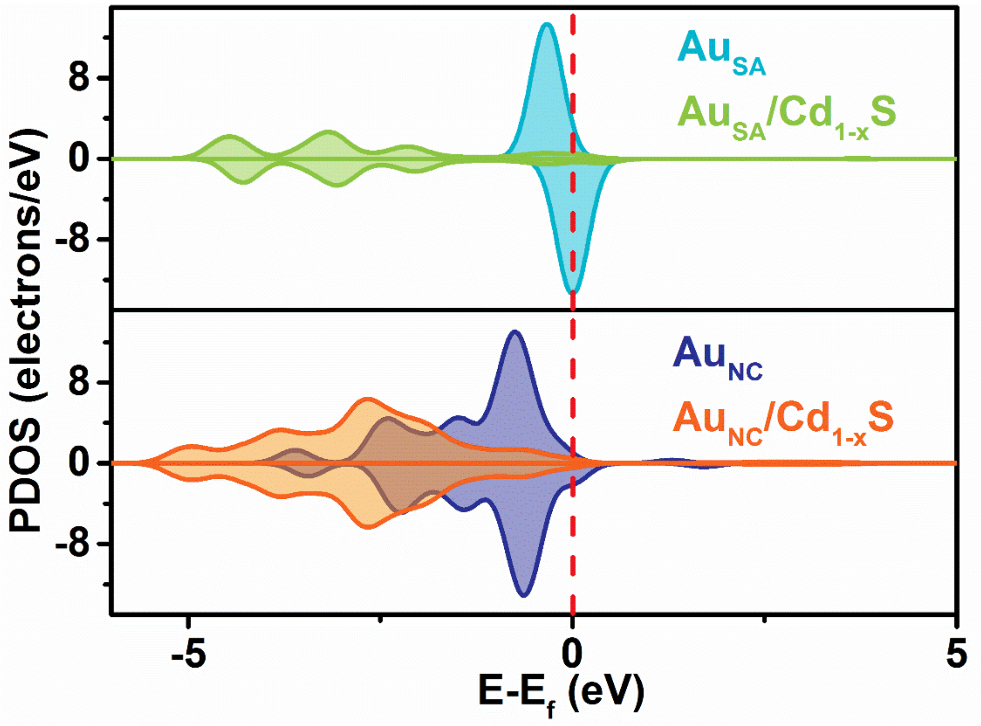


**Supplementary Fig. 27** PDOS of Au 5d orbits in $Au_{SA}$, $Au_{SA}/Cd_{1-x}S$, $Au_{NC}$ and $Au_{NC}/Cd_{1-x}S$.

**28. TR-PL spectra of $Cd_{1-x}S$, $Au_{SA}/Cd_{1-x}S$ and $Au_{NC}/Cd_{1-x}S$ samples**

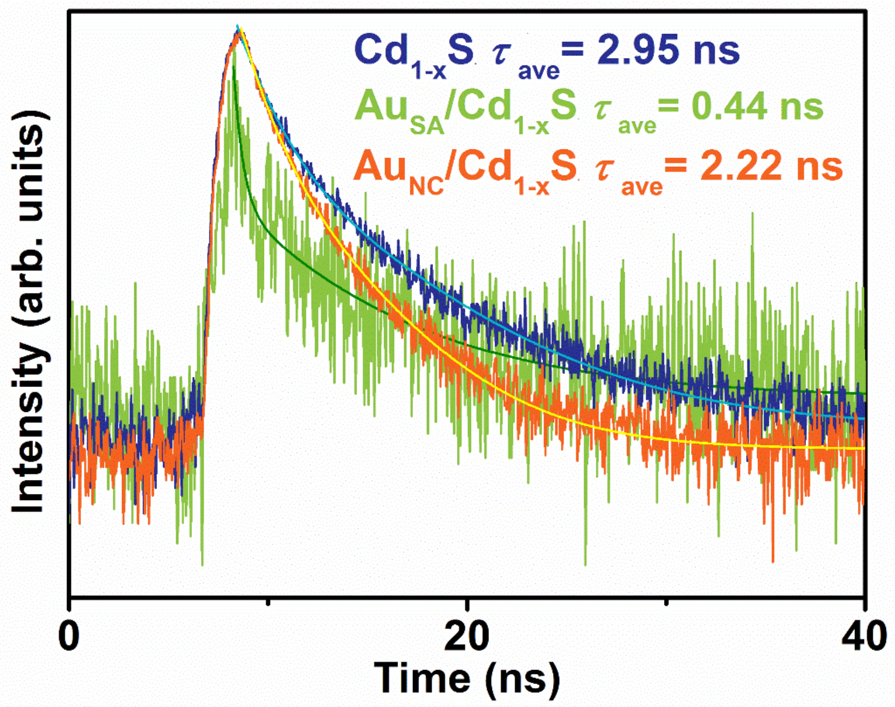


**Supplementary Fig. 28** TR-PL spectra of $Cd_{1-x}S$, $Au_{SA}/Cd_{1-x}S$ and $Au_{NC}/Cd_{1-x}S$ samples.

## 29. Band structure of CdS and $Cd_{1-x}S$ system

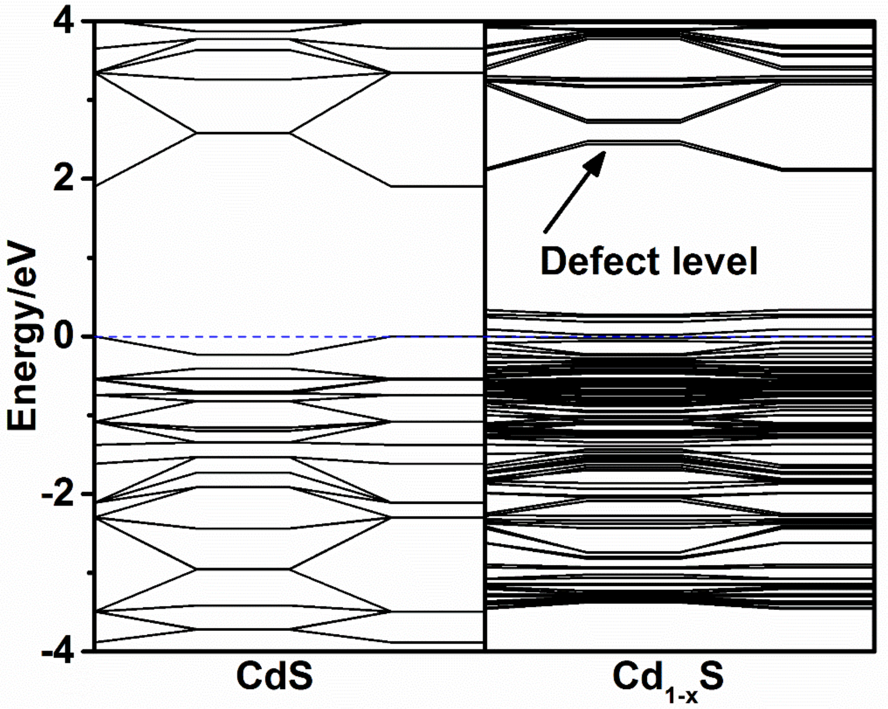


**Supplementary Fig. 29** Band structure of CdS and $Cd_{1-x}S$.

## 30. Transient photocurrent response and SPV spectra of $CdS_{1-x}$ and Au/$CdS_{1-x}$

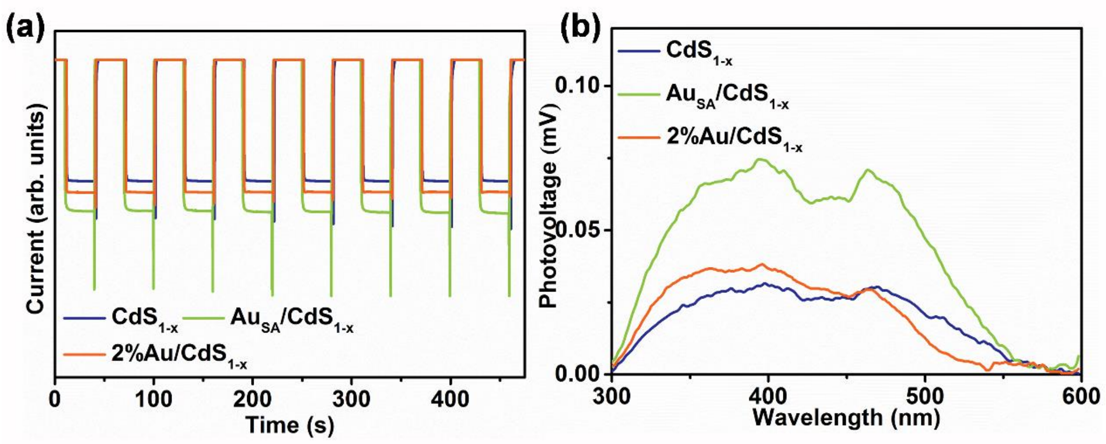


**Supplementary Fig. 30** Transient photocurrent response (a) and SPV spectra (b) of $CdS_{1-x}$, $Au_{SA}/CdS_{1-x}$ and 2%Au/$CdS_{1-x}$.

## 31. Global reaction profiles for $CO_2$ reduction in $Cd_{1-x}S$ system

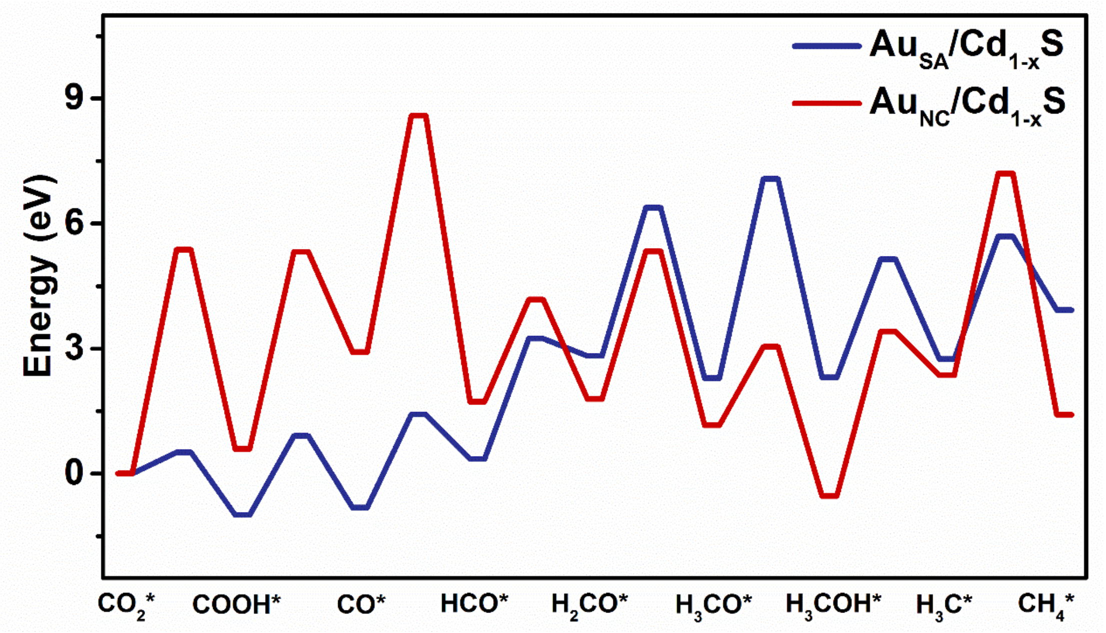


**Supplementary Fig. 31** Global reaction profiles for $CO_2$ reduction on $Au_{SA}/Cd_{1-x}S$ and $Au_{NC}/Cd_{1-x}S$.

## 32. Elemental composition

**Supplementary Table 1** Atomic percentage of samples.

| Sample | Cd (%) | S (%) | Cd : S |
|---|---|---|---|
| $Cd_{1-x}S$ | 43.02 | 56.98 | 0.76 : 1 |
| $CdS_{1-x}$ | 57.84 | 42.16 | 1 : 0.73 |

## 33. Au coordination environments determined from the analysis of EXAFS spectra

**Supplementary Table 2** A summary of Au coordination environments determined from the analysis of EXAFS spectra.

| Sample | Au-S distance (Å) | CN (S) | $\sigma^2$ ($10^{-3}$ Å$^2$) | $\delta E_0$ (eV) | ρ (%) |
|---|---|---|---|---|---|
| $Au_{SA}/Cd_{1-x}S$ | 2.27±0.02 | 2.2±0.4 | 0.3±2.1 | 5.8±2.0 | 1.3 |

## 34. Formation energy of single Au atoms loaded $Cd_{1-x}S$ surface

**Supplementary Table 3** Formation energy of single Au atoms loaded $Cd_{1-x}S$ surface with different sites (Unit: eV).

| Site | Surface S atoms | Cd vacancy |
|---|---|---|
| $E_f$ | -1.89 | -5.30 |

## 35. Recent improvement of photocatalytic conversion of $CO_2$

**Supplementary Table 4** Recent improvement of photocatalytic conversion of $CO_2$ with $H_2O$.

| Systems | Photocatalysts | Conditions | Products (μmol $g^{-1}$ $h^{-1}$) | Reference |
|---|---|---|---|---|
| Gas-solid (water vapor) | Au@CdS/$TiO_2$ | 300 W Xe lamp (320-780 nm; 100 mW/$cm^2$);<br>Irradiation time: 2 h | CO: 18.2<br>$CH_4$: 41.6<br>$H_2$: 0.6 | 1 |
| | Pt@CdS/$TiO_2$ | 300 W Xe lamp (320-780 nm; 100 mW/$cm^2$);<br>Irradiation time: 2 h | CO: 0.7<br>$CH_4$: 36.8<br>$H_2$: 16.2 | 2 |
| | Ru/CdS | 375 W Xe lamp and IR lamp (710 mW/$cm^2$);<br>Irradiation time: 4 h | CO: 0.1<br>$CH_4$: 2.6 | 3 |
| | $CuIn_5S_8$ with S vacancies | 300 W Xe lamp (420-780 nm; 50 mW/$cm^2$);<br>Irradiation time: 24 h | $CH_4$: 8.7 | 4 |
| | $SnS_2$ | 300 W Xe lamp (420-780 nm; 50 mW/$cm^2$);<br>Irradiation time: 20 h | CO: 12.3 | 5 |
| | Pt/$TiO_2$-$SiO_2$ | 300 W Xe lamp (420-780 nm);<br>Irradiation time: 4 h | $CH_4$: 9.7<br>$H_2$: 58.7 | 6 |
| | $Au_{SA}$/$Cd_{1-x}S$ | 300 W Xe lamp (200-800 nm; 600 mW/$cm^2$);<br>Irradiation time: 8 h | CO: 32.2<br>$CH_4$: 11.3<br>$H_2$: 7.9 | This work |
| Gas-liquid-solid | CdS QDs/$ZnIn_2S_4$ | 300 W Xe lamp (420-800 nm);<br>Sacrificial agent: TEOA;<br>Cocatalyst: $Co(bpy)_3^{2+}$;<br>Irradiation time: 0.5 h | CO: 2182.5 | 7 |
| | Au@CdS | 300 W Xe lamp (420-800 nm);<br>Sacrificial agent: TEOA;<br>Cocatalyst: $Co(bpy)_3^{2+}$;<br>Irradiation time: 1 h | CO: 3758.0 | 8 |
| | BCN/CdS | 300 W Xe lamp (420-800 nm);<br>Sacrificial agent: TEOA;<br>Cocatalyst: $Co(bpy)_3^{2+}$;<br>Irradiation time: 1.5 h | CO: 250.0 | 9 |
| | CdS/Co-EDTA | 300 W Xe lamp (420-800 nm);<br>Irradiation time: 6 h | CO: 392.0<br>$H_2$: 1844.0 | 10 |

## 36. Adsorption energy of $CO_2$

**Supplementary Table 5** The adsorption energy of $CO_2$ on the surface of $Cd_{1-x}S$ and $CdS_{1-x}$ system (Unit: eV).

| Configuration | Site | $E_{ads}$ |
|---|---|---|
| $Cd_{1-x}S$ | Vacancy | -0.43 |
| $Au_{SA}/Cd_{1-x}S$ | Au | -0.32 |
| | Vacancy | -0.76 |
| $Au_{NC}/Cd_{1-x}S$ | Au | -0.43 |
| | Vacancy | -0.42 |
| $CdS_{1-x}$ | Vacancy | -0.15 |
| $Au_{SA}/CdS_{1-x}$ | Au | -0.22 |
| | Vacancy | -0.09 |
| $Au_{NC}/CdS_{1-x}$ | Au | -0.20 |
| | Vacancy | -0.19 |

## 37. Experiments

**Materials:** $HAuCl_4 \cdot 3H_2O$ (99.9%) and $CdCl_2 \cdot 5H_2O$ (99.95%) were purchased from Shanghai Aladdin Chemical Reagent Co., Ltd. $NaS_2 \cdot 9H_2O$ (98%) and hydrochloric acid (HCl, 36%) were purchased from Chengdu Kelong Chemical Co., Ltd. All of the chemicals were without any further purification before the experiment. Deionized water was used in the whole experiment.

**Characterization:** X-ray diffraction (XRD) patterns were recorded on DX-2700 powder diffractometer with Cu $K_\alpha$ radiation operated at 40 kV/40 mA. The microstructures of $Cd_{1-x}S$ and $Au_{SA}/Cd_{1-x}S$ were obtained by transmission electron microscopy (TEM) and high-resolution transmission electron microscopy (HRTEM, Tecnai G2 F30). The TEM and HRTEM images of $Au_{NC}/Cd_{1-x}S$ were carried out on JEOL JEM 2100F operated at 200 kV with EDX mapping. Electron paramagnetic resonance (EPR) spectra were measured on an electron spin resonance spectrometer (JES-FA200) operating at room temperature. Aberration-corrected high-angle annular dark-field scanning transmission electron microscopy (HAADF-STEM) was carried out on the fifth order aberration-corrected transmission electron microscope (JEOL ARM200CF). UV-vis spectra were measured on the spectrophotometer (Shimadzu UV-2600) equipped with an integrating sphere using $BaSO_4$ as the reflectance standard reference at room temperature. X-ray photoelectron spectrometer (Thermo ESCALAB250Xi) was employed to detect the X-ray photoelectron spectrum (XPS). The $N_2$ sorption isotherm was measured on Quadrasorb SI. The surface photovoltage (SPV) spectrum was recorded on a monochromator (Omni-5007). The time-resolved fluorescence emission (TR-PL) spectrum was performed on FLS1000 fluorescence spectrometer (Excitation wavelength: 405 nm).

**X-ray absorption measurement and data analysis:** X-ray absorption spectra (XANES and EXAFS) at the Cd K absorption edge were recorded at the P65 beamline of the PETRA III synchrotron radiation source (DESY, Hamburg, Germany) in transmission mode using ionization chambers as detectors. The energy of the X-ray photons was further selected by a Si (311) double-crystal monochromator and higher harmonics were rejected using a Pt-coated Si mirror. The spectra were normalized and the extended X-ray absorption fine structure spectra (EXAFS) background subtracted using the ATHENA program from the IFFEFIT software package[11]. The $k^1$-weighted EXAFS functions were Fourier transformed (FT) in the k range of 2.5-12.0 $Å^{-1}$ and multiplied by a Hanning window with sill size of 1 $Å^{-1}$. The experimental spectra were fitted to a wurtzite CdS structural model as reported in the Inorganic Crystal Structure Database

(ICSD, collection code 154186). The fits of the EXAFS data were performed on the $k^1$- and $k^2$-weighted data using Artemis by a least square method in R-space between 1.2 and 2.4 Å[7]. X-ray absorption spectra at the Au $L_3$ absorption edge were recorded at the Cat-Act beamline of the KIT synchrotron radiation source (Karlsruhe, Germany) in fluorescence mode using a single element silicon drift detector (1 mm thickness, Vortex, Hitachi). The energy of the X-ray photons was further selected by a Si (111) double-crystal monochromator. The spectra were normalized and the extended X-ray absorption fine structure spectra (EXAFS) background subtracted using the ATHENA program from the IFFEFIT software package[11]. The $k^1$-weighted EXAFS functions were Fourier transformed (FT) in the k range of 2.0-10.0 Å$^{-1}$ and multiplied by a Hanning window with sill size of 1 Å$^{-1}$. Then the amplitude reduction factor $S_0^2$=0.79 was obtained by fitting the Au foil reference spectrum. The experimental spectra were fitted to a $Au_2S$ structural model as reported in the *Inorganic Crystal Structure Database* (*ICSD, collection code 78718*). The fits of the EXAFS data were performed on the $k^1$- and $k^2$-weighted data using Artemis by a least square method in R-space between 1.0 and 2.3 Å[11]. Coordination numbers, interatomic distances, energy shift ($\delta E_0$) and mean square deviation of interatomic distances ($\sigma^2$) were refined during fitting. The absolute misfit between theory and experiment was expressed by ρ.

**Photoelectrochemical test:** Transient photocurrent response was investigated with CHI660E workstation by using Pt wire as the counter electrode, and saturated calomel electrode (SCE) as the reference electrode. The experiment was employed at the 300W Xe lamp. The working electrode with an active area of 1 $cm^2$ was prepared by a coating technique (FTO, film thickness: 50 μm). The photoelectrochemical measurements were performed in the 1.2 M $Na_2SO_3$ solution.

## 38. Calculation method and model

Density functional theory (DFT) was employed in the whole calculations based on Materials Studio software with the Cambridge Sequential Total Energy Package (CASTEP) code[12]. The generalized gradient approximation (GGA) with the Perdew-Burke-Ernzerhof (PBE) functional was carried out[13,14]. The cutoff energy was set to be 380 eV. According to the convergence test in Supplementary Fig. 32, the k-point was set to be 2×2×1. The CdS surface was cleaved along the (001) orientation of bulk CdS. Accoding to the convergence test in Supplementary Fig. 33, a 4×4 supercell was constructed, which involved 16 Cd atoms and 16 S atoms.

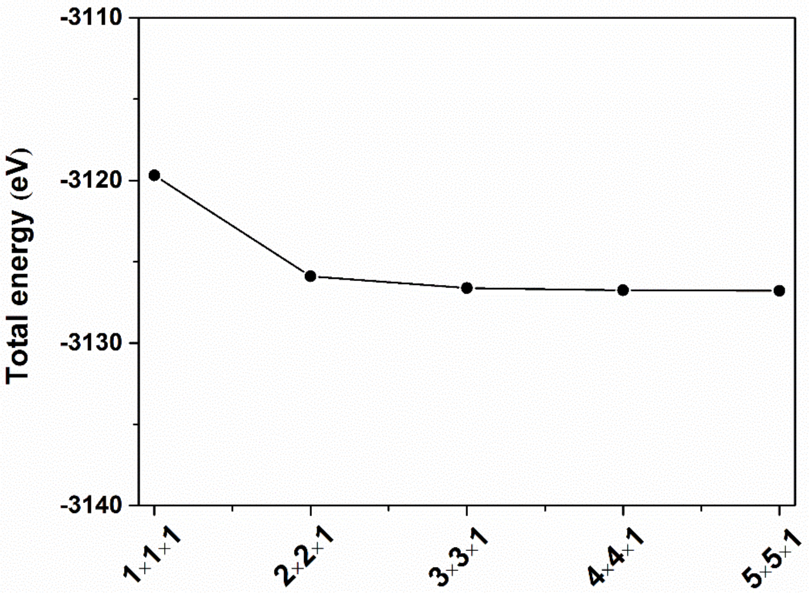


**Supplementary Fig. 32** Convergence test of k-point.

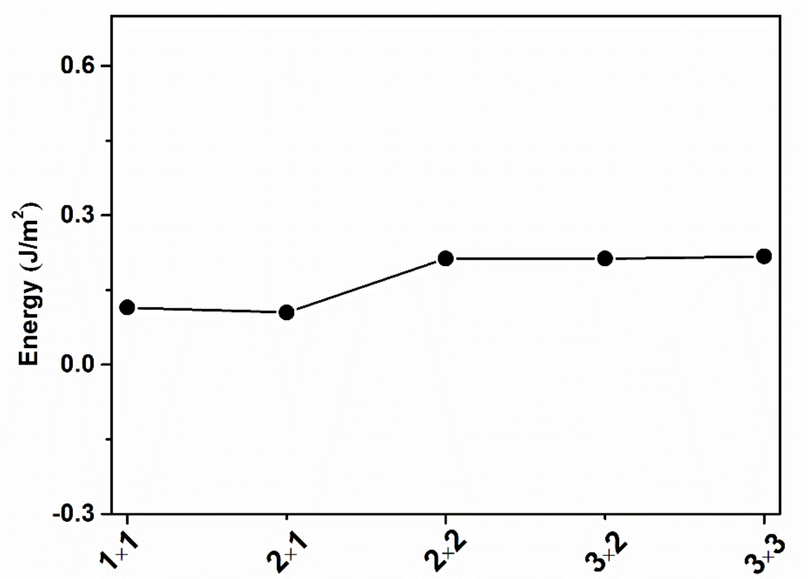


**Supplementary Fig. 33** Convergence test of surface energy.

## 39. References


1. Wei, Y., *et al.* Fabrication of inverse opal $TiO_2$-supported Au@CdS core-shell nanoparticles for efficient photocatalytic $CO_2$ conversion. *Appl. Catal. B: Environ.* **179**, 422-432 (2015).
2. Wei, Y., *et al.* 3D ordered macroporous $TiO_2$-supported Pt@CdS core-shell nanoparticles: design, synthesis and efficient photocatalytic conversion of $CO_2$ with water to methane. *J. Mater. Chem. A* **3**, 11074-11085 (2015).
3. Cai, S., Zhang, M., Li, J., Chen, J., Jia, H. Anchoring single-atom Ru on CdS with enhanced $CO_2$ capture and charge accumulation for high selectivity of photothermocatalytic $CO_2$ reduction to solar fuels. *Solar RRL* **5**, 2000313 (2021).
4. Li, X., *et al.* Selective visible-light-driven photocatalytic $CO_2$ reduction to $CH_4$ mediated by atomically thin $CuIn_5S_8$ layers. *Nat. Energy* **4**, 690-699 (2019).
5. Jiao, X., *et al.* Partially oxidized $SnS_2$ atomic layers achieving efficient visible-light-driven $CO_2$ reduction. *J. Am. Chem. Soc.* **139**, 18044-18051 (2017).
6. Dong, C., *et al.* Size-dependent activity and selectivity of carbon dioxide photocatalytic reduction over platinum nanoparticles. *Nat. Commun.* **9**, 1252 (2018).
7. Zhu, Z., *et al.* A hierarchical heterostructure of CdS QDs confined on 3D $ZnIn_2S_4$ with boosted charge transfer for photocatalytic $CO_2$ reduction. *Nano Res.* **14**, 81-90 (2021).
8. Zhang, P., Wang, S., Guan, B. Y., Lou, X. W. Fabrication of CdS hierarchical multi-cavity hollow particles for efficient visible light $CO_2$ reduction. *Energ. Environ. Sci.* **12**, 164-168 (2019).
9. Zhou, M., Wang, S., Yang, P., Huang, C., Wang, X. Boron carbon nitride semiconductors decorated with CdS nanoparticles for photocatalytic reduction of $CO_2$. *ACS Catal.* **8**, 4928-4936 (2018).
10. Zhao, G., *et al.* Efficient photocatalytic $CO_2$ reduction over Co(II) species modified CdS in aqueous solution. *Appl. Catal. B: Environ.* **226**, 252-257 (2018).
11. Ravel, B., Newville, M. ATHENA, ARTEMIS, HEPHAESTUS: data analysis for X-ray absorption spectroscopy using IFEFFIT. *J. Synchrotron Radiat.* **12**, 537-541 (2005).
12. Segall, M. D., *et al.* First-principles simulation: ideas, illustrations and the CASTEP code. *J. Phys.: Condens. Mat.* **14**, 2717-2743 (2002).
13. Perdew, J. P., Burke, K., Ernzerhof, M. Generalized gradient approximation made simple. *Phys. Rev. Lett.* **77**, 3865-3868 (1996).
14. White, J. A., Bird, D. M. Implementation of gradient-corrected exchange-correlation potentials in car-parrinello total-energy calculations. *Phys. Rev. B* **50**, 4954-4957 (1994).
